# Germline rare variants in HER2-positive breast cancer predisposition: a systematic review and meta-analysis

**DOI:** 10.3389/fonc.2024.1395970

**Published:** 2024-06-24

**Authors:** Angelica Cerveira de Baumont, Nathan Araujo Cadore, Luana Giongo Pedrotti, Giovana Dallaio Curzel, Jaqueline Bohrer Schuch, Marina Bessel, Cláudia Bordignon, Mahira Lopes Rosa, Gabriel de Souza Macedo, Daniela Dornelles Rosa

**Affiliations:** ^1^ Responsabilidade Social, Hospital Moinhos de Vento, Porto Alegre, RS, Brazil; ^2^ Programa de Pós-Graduação em Genética e Biologia Molecular, Universidade Federal do Rio Grande do Sul, Porto Alegre, Brazil; ^3^ Programa de Pós-Graduação em Ciências Médicas, Universidade Federal do Rio Grande do Sul, Porto Alegre, Brazil

**Keywords:** Hereditary breast cancer, HER2+, pathogenic variants, *TP53*, *BRCA*

## Abstract

**Introduction:**

Approximately 10% of breast cancer (BC) cases result from hereditary causes. Genetic testing has been widely implemented in BC care to determine hereditary cancer syndromes and personalized medicine. Thus, identification of individuals carrying germline pathogenic variants could be useful to provide appropriate prophylactic or screening measures for each BC subtype, however, there are few formal recommendations for genetic testing in this sense so far. In this study, we assessed rare germline variants in a specific group of genes in order to determine the association with human epidermal growth factor 2 enriched (HER2+) BC phenotype through a systematic review and meta-analysis comparing subtypes overexpressing HER2 with other clinically recognized subtypes of BC. This review was registered with PROSPERO (ID: CRD42023447571).

**Methods:**

We conducted an online literature search in PubMed (MEDLINE), Scopus, and EMBASE databases. We included original studies that investigated germline variants in HER2+ BC patients and selected the studies that reported only rare and/or pathogenic germline variants. We assessed the risk of bias and quality of the studies using the Joanna Briggs Institute Critical Appraisal checklists and the Modified Newcastle-Ottawa Scale for Genetic Studies, respectively. Considering hormone receptor and HER2 expression status, we compared gene-based risks initially in HR-HER2-, HR+HER2-, HR+HER2+, and HR-HER2+ groups, conducting separate meta-analyses using the random effects model for each comparison, and within them for each gene.

**Results:**

Of the total 36 studies describing germline variants, 11 studies provided information on the prevalence of variants in the different clinically relevant BC subtypes and allowed comparisons. Germline variants within eight genes showed significant differences when meta-analyzed between the BC groups: *BRCA1*, *BRCA2*, *TP53*, *ATM*, *CHEK2*, *PALB2*, *RAD51C*, and *BARD1*. Notably, *TP53*, *ATM*, and *CHEK2* germline variants were identified as predisposing factors for HER2+ subtypes, whereas *BRCA1*, *BRCA2*, *PALB2*, *RAD51C*, and *BARD1* germline variants were associated with a predisposition to low HER2 expression. Main concerns about bias and quality assessment were the lack of confounding factors control; and comparability or outcome assessment, respectively.

**Discussion:**

Our findings underscore the connection between germline variants and differential expression of the HER2 protein and BC subtypes.

**Systematic review registration:**

https://www.crd.york.ac.uk/PROSPERO, identifier CRD42023447571.

## 1 Introduction

Breast cancer (BC) was the most frequent tumor and the leading cause of cancer-related deaths among women, with more than 2,3 million cases worldwide in 2022 (1). BC is highly heterogeneous at morphologic and molecular levels, which directly impacts the disease prognosis and treatment (2). Four main intrinsic BC subtypes have been well-characterized and comprise the vast majority of the biological diversity in BC: Luminal A, Luminal B, human epidermal growth factor 2 (HER2) enriched (HER2+), and Basal-like (3). These subtypes differ regarding the proliferation/cell cycle-related and luminal/hormone-regulated pathways, gene expression regulation mechanisms, such as methylation, and mutational burden (4).

HER2, also known as Neu or ErbB2, belongs to the ERBB receptor family, which is expressed in many epithelial, mesenchymal, and neuronal cells, and has different roles in cellular development, proliferation, and differentiation (5). The HER2 protein composes a heterodimer with other ERBB and epidermal growth factor receptors (EGFRs). These complexes recognize ligand hormones and trigger subsequent signal transduction, activating downstream signaling pathways such as PI3K-AKT and MEK-ERK (6). HER2 overexpression is a negative prognostic factor and accounts for about 15–20% of all BC cases. One of the main mechanisms of HER2 activation is the amplification of the encoding gene *ERBB2*, leading to HER2 overexpression (7). This overexpression can be identified through the immunohistochemistry status (IHC3+), or *in situ* hybridization (ISH) measurement of an *ERBB2* gene copy number of six or more, or through the *ERBB2*/CEP17 (centromeric region of chromosome 17) ratio of 2.0 or greater (8).

Most BC cases (approximately 80%) are not metastatic at the time of diagnosis. For these patients, the treatment strategy is based on tumor eradication and recurrence prevention (9). Hence, choosing effective drugs is essential for treatment success. Specific therapeutic strategies (both neoadjuvant and adjuvant therapies) were established based on BC molecular subtypes. For example, monoclonal antibodies (trastuzumab and pertuzumab) associated with conventional chemotherapeutic drugs are effective in the treatment of HER2+ BC patients (10). Although cancer therapeutic schemes are based on cancer staging and molecular subtypes, there is a broad spectrum of treatment responses, which may be influenced by other factors, such as genetic and epigenetic alterations.

In the last decades, several studies reported the landscape of germline variants in cancer predisposition genes as well as their impact on cancer risk. As an example, Li-Fraumeni syndrome (LFS), an autosomal dominantly inherited condition, is a rare hereditary cancer syndrome characterized by a high and early-onset cancer risk caused by pathogenic variants (PVs) in the tumor suppressor gene *TP53* (11). Other well-known genetic alterations include PVs or likely pathogenic variants (LPVs) in the *BRCA1* and *BRCA2* genes, linked to hereditary breast and ovarian cancer syndrome (12). Moreover, these germline variants may be associated with a poorer BC prognosis (13, 14).

Even though genomic studies have characterized the germline architecture of BC patients (15, 16), little is known regarding the impact of these variants on the predisposition and prognosis of specific BC subtypes. Thus, this study aimed to perform a comprehensive assessment of rare germline variants associated with HER2+ BC. To this end, we conducted a systematic review and meta-analysis of genomic, exomic, and panel sequencing studies that assessed germline variants associated with the prediction of HER2+ BC, in comparison to other BC subtypes.

## 2 Methods

### 2.1 Search approach

This systematic review and meta-analysis was registered with the International Prospective Register of Systematic Reviews (PROSPERO; registration ID: CRD42023447571). We adhered to the PRISMA (Preferred Reporting Items for Systematic Reviews and Meta-Analyses) checklist to ensure transparency and consistency in the presentation of data (**Supplementary Tables 1, 2**). Online literature search was performed in the PubMed (MEDLINE), Scopus, and EMBASE databases in order to collect eligible studies until July 07, 2023. The search was conducted based on three concepts (1): breast cancer (2); HER2; and (3) genetic variation. The search terms are detailed in the **Supplementary Material**. In addition, reference lists, including systematic reviews, meta-analyses, and original articles, were examined to identify relevant studies. All retrieved results were exported to the Rayyan tool (17), where duplications were removed and the remaining studies were evaluated.

### 2.2 Selection of studies

On initial selection, we included studies that satisfied the following criteria (1): original studies that enrolled patients with histologically confirmed HER2+ BC (2); studies evaluating germline variants associated with HER2+ BC prediction and prognosis (3); English language. We excluded (1): reviews and meta-analysis (2); *in vitro* studies (3); animal models (4); insufficient data; and (5) gray literature. Two reviewers independently evaluated the retrieved studies that meet the predetermined criteria, and a third reviewer was consulted in case of controversy.

In a second step, we selected the studies that reported only rare and/or pathogenic variants. The criteria applied by each study to classify the variants’ pathogenicity are described in **Supplementary Table 3**. We chose this focus because these types of germline variants have a greater impact on BC. Rare loss-of-function and pathogenic variants are known to be responsible for hereditary cancer. Moreover, the studies reporting common variants are heterogeneous and pose a challenge for data standardization in the analysis.

### 2.3 Data extraction

The following information was independently extracted from all the included reports: first author, study type, year of publication, nation, sample size, population, identified pathogenic variants, outcomes in the studied groups, and genomic assay. Any discrepancies during the process were resolved by discussion with the third reviewer. In cases where the data were incompletely described in an article, we contacted the corresponding author to request the data.

### 2.4 Risk of bias and quality assessment

We assessed the risk of bias in all the included studies concerning their design, conduct, and analysis, using the Joanna Briggs Institute Critical Appraisal checklists tailored to cross-sectional, case-control, or cohort studies (18). Each item of the instrument was categorized as yes/no/unclear for each potential bias. For the overall assessment, each study was assessed for inclusion, exclusion, or need for further information.

We used the Modified Newcastle-Ottawa Scale for Genetic Studies (19) to assess the quality of the studies. The studies were rated with 0–8 stars based on the scale items: studies with at least 6 stars were considered to be of high quality, studies with 3–5 stars were considered to be of fair quality, and studies with less than 2 stars were considered to be of inferior quality.

### 2.5 Statistical methods

A meta-analysis was performed using the random effects model. Odds ratios (OR) were estimated along with their respective 95% confidence intervals (CI). Given the clinical significance of hormone receptor (HR) and HER2 expression status, the comparison was performed based on gene variants between the following groups: HR-HER2- (triple-negative BC, TNBC), HR+HER2-, HR+HER2+, and HR-HER2+. We carried out a different meta-analysis for each comparison (HR+HER2+ *versus* HR-HER2+; HR+HER2+ *versus* HR+HER2-; HR+HER2+ *versus* TNBC; HR-HER2+ *versus* HR+HER2-; HR-HER2+ *versus* TNBC and HR+HER2- *versus* TNBC) and within them for each gene (*ATM*, *BARD1, BRCA1*, *BRCA2*, *CHEK2*, *PALB2*, *RAD51C*, and *TP53*, when possible). Only genes that were reported in two distinct studies, with a minimum of one documented genetic variant, were considered for inclusion in the analysis. In addition, prediction intervals were presented. Heterogeneity between studies was evaluated using Cochran’s Q test and the inconsistency index (I²). All analyses were performed using R software version 4.1.3 (20) with the meta package version 7.0-0 (21).

## 3 Results

### 3.1 Characteristics of the included studies

**Figure 1:**
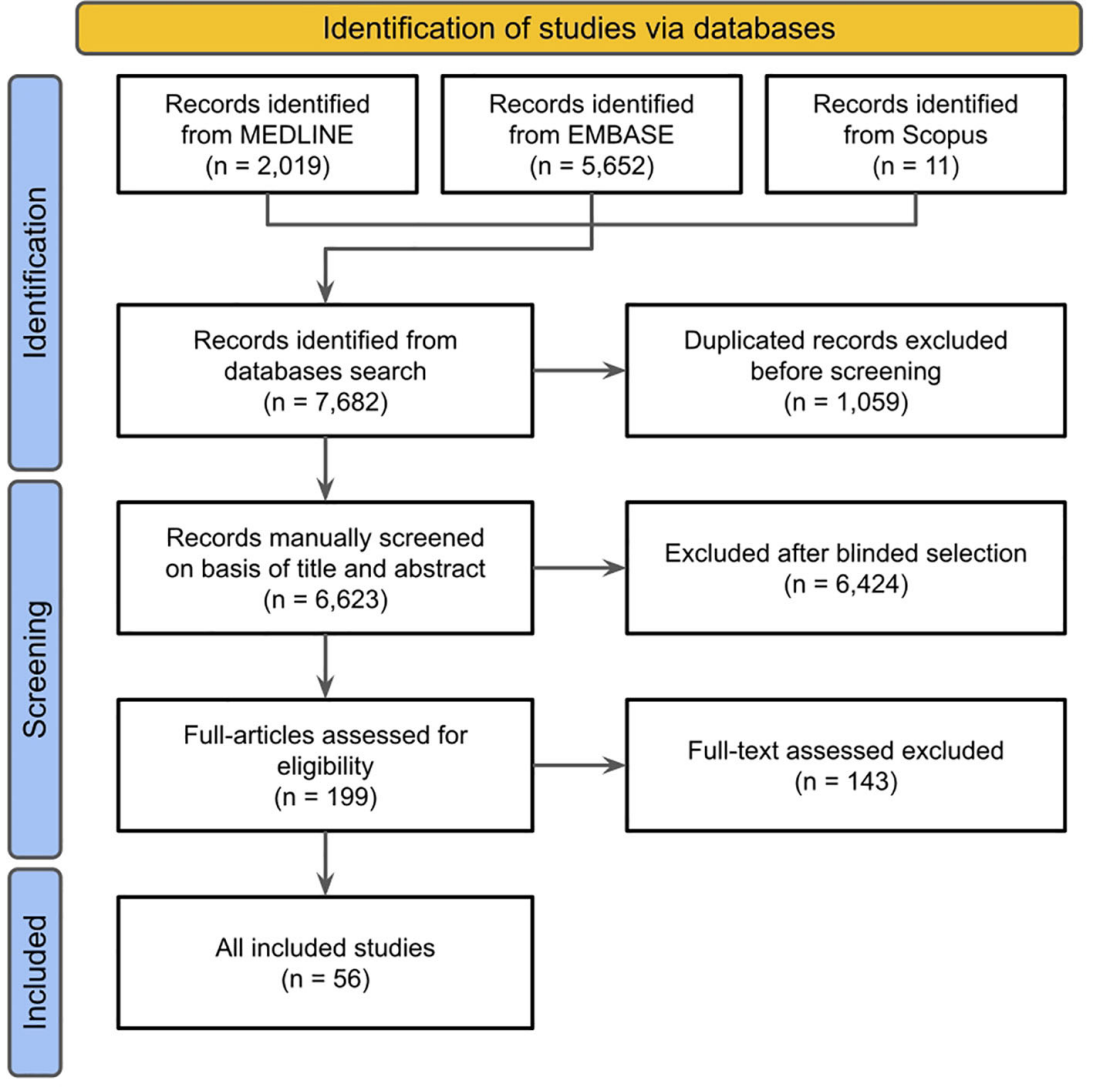
PRISMA diagram of study selection strategy from literature search results.

The search strategy across three different databases retrieved 6,623 records, from which 56 studies were selected by inclusion criteria (**Figure 1**). The included studies performed methodologies for genomic analyses that included whole-genome sequencing, whole-exome sequencing, and gene panel testing using next-generation sequencing technology. First, in order to group comparable data, studies were classified into rare or common variants depending on the types of genetic variants reported. A total of 36 studies reported rare single-nucleotide variants (SNVs), insertion-deletion variants, or copy number alterations. Of these, 32 were eligible for data extraction. Most studies were from Western European and East Asian groups, followed by North American, Latin American, South Asian, and Middle Eastern. The lower representation of specific global regions is also reflected in the populations encountered in the study. No study included African populations, and only two were conducted with the Latin American population. The information about the studied population, design, genomic assessment method, genes, and variants classification considered for analysis were described in **Supplementary Table 4**.

The genes mainly reported were *BRCA1* and *BRCA2*, followed by *TP53*, *PALB2*, and *ATM* (**Supplementary Figure 1**). Only six studies were case-control studies conducted with a health control cohort (individuals without BC). The heterogeneity in the classification of study groups and insufficient data posed challenges for comparisons when considering BC subtypes and receptor expression. A total of 13 studies reported only the information of total BC cases and did not stratify regarding HR or HER2 status, and were not included in the statistical analyses (**Supplementary Table 4**). The risk based on genes carrying a germline variant was initially compared in the groups (TNBC, HR+HER2-, HR+HER2+, and HR-HER2+). Altogether, 11 studies provided information on the prevalence of germline variants among these groups and allowed comparisons.

### 3.2 Risk of bias and quality assessment

The risk of bias was assessed for the 11 publications included in the meta-analysis using the Joanna Briggs Institute (JBI) Critical Appraisal Tools. According to the JBI tools, all studies were considered suitable for inclusion in this review. The most common concerns regarding risk of bias were due to failure to identify and control for confounding factors (**Supplementary Figures 2–4**).

All studies included in the meta-analysis were considered of high or fair quality (3 or more stars) on the modified Newcastle-Ottawa scale for Genetic Studies. The most common concerns were on comparability and outcome assessment (**Supplementary Table 5**).

### 3.3 Meta-analysis of germline variants among BC subtypes

A meta-analysis comparing the predisposition between BC subtypes was performed pair-by-pair. A total of nine studies (22–30) presented frequencies of germline variants that allowed the following comparisons: HR-HER2+ *versus* TNBC (**Supplementary Figure 5**), HR-HER2+ *versus* HR+HER2- (**Supplementary Figure 6**), HR+HER2+ *versus* TNBC (**Supplementary Figure 7**), HR+HER2+ *versus* HR+HER2- (**Supplementary Figure 8**), and HR+HER2+ *versus* HR-HER2+ (**Supplementary Figure 9**). Moreover, eleven studies (22–32) allowed the comparison of HR+HER2- *versus* TNBC (**Supplementary Figure 10**). For each comparison, genes were included when a variant was reported in at least two studies. The genes included in each analysis are reported in **Table 1**.

**Table 1:** Genes included in comparative meta-analysis of breast cancer subtypes.

Comparison	Genes
HR-HER2+ *versus* TNBC	*BRCA1*, *BRCA2*, *PALB2*, *TP53*, *ATM*, *CHEK2*, *RAD51C*, *BARD1*, *BRIP1*, *MSH6*, and *NBN*.
HR-HER2+ *versus* HR+HER2-	*BRCA1*, *BRCA2*, *PALB2*, *TP53*, *ATM*, *CHEK2*, *BRIP1*, *MSH6*, *RAD51D*, and *MUTYH*.
HR+HER2+ *versus* TNBC	*BRCA1*, *BRCA2*, *PALB2*, *TP53*, *ATM*, *CHEK2*, *RAD51C*, *BRIP1*, and *NBN*.
HR+HER2+ *versus* HR+HER2-	*BRCA1*, *BRCA2*, *PALB2*, *TP53*, *ATM*, *CHEK2*, *BRIP1*, *RAD51D*, *NBN*, and *FANCM*.
HR+HER2+ *versus* HR-HER2+	*BRCA1*, *BRCA2*, *PALB2*, *TP53*, *ATM*, *CHEK2*, *BRIP1*, *MSH6*, *MUTYH*, and *NBN*.
HR+HER2- *versus* TNBC	*BRCA1*, *BRCA2*, *PALB2*, *TP53*, *ATM*, *CHEK2, RAD51C, BRIP1*, *BARD1*, *PMS2*, *MSH6*, *RAD51D*, *NBN*, *FANCM*, *FANCD2*.

**Figure 2:**
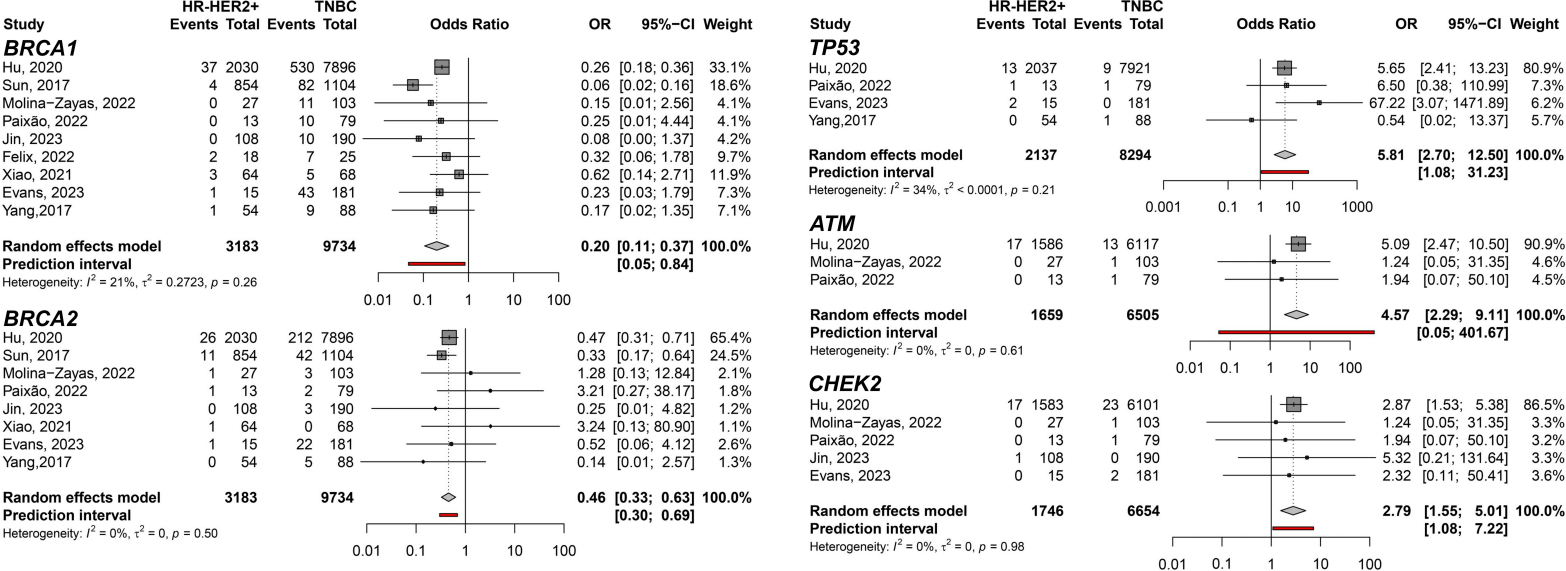
Forest plots showing the predisposing of HR-HER2+ (cases) breast cancer subtype compared to TNBC (controls) for variant carriers on genes *BRCA1*, *BRCA2*, *TP53*, *ATM*, and *CHEK2*.

**Figure 3:**
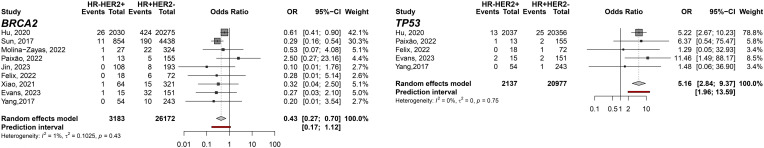
Forest plots of the comparison of HR-HER2+ (cases) breast cancer subtype predisposition related to HR+HER2- (controls) for the genes *BRCA2* and *TP53*.

First, when the predisposition to HR-HER2+ was evaluated comparing to TNBC, germline variants on *TP53* gene showed the highest pooled Odds Ratio (pOR) of developing HR-HER2+ BC among the meta-analyzed genes (pOR: 5.81; 95% CI: 2.70–12.50; n=10,431). Moreover, the pOR for *ATM* was 4.57 (95% CI 2.29–9.11; n=8,164), and for *CHEK2* was 2.79 (95% CI: 1.55–5.01; n=8,400). Germline variants present in *BRCA1* (pOR: 0.20; 95% CI 0.11–0.37; n=12,917) and *BRCA2* (pOR: 0.46; 95% CI 0.33–0.63; n=12,917) were found to predispose TNBC compared to HR-HER2+ (**Figure 2**). Interestingly, when HR-HER2+ was compared to HR+HER2-, *TP53* germline variants were also found to predispose HR-HER2+ (pOR: 5.16; 95% CI: 2.84–9.32; n=23,114) while *BRCA2* germline variants carriers were found to predispose HR+HER2- subtype (pOR: 0.43; 95% CI: 0.27; 0.70; n=29,355) (**Figure 3**).

**Figure 4:**
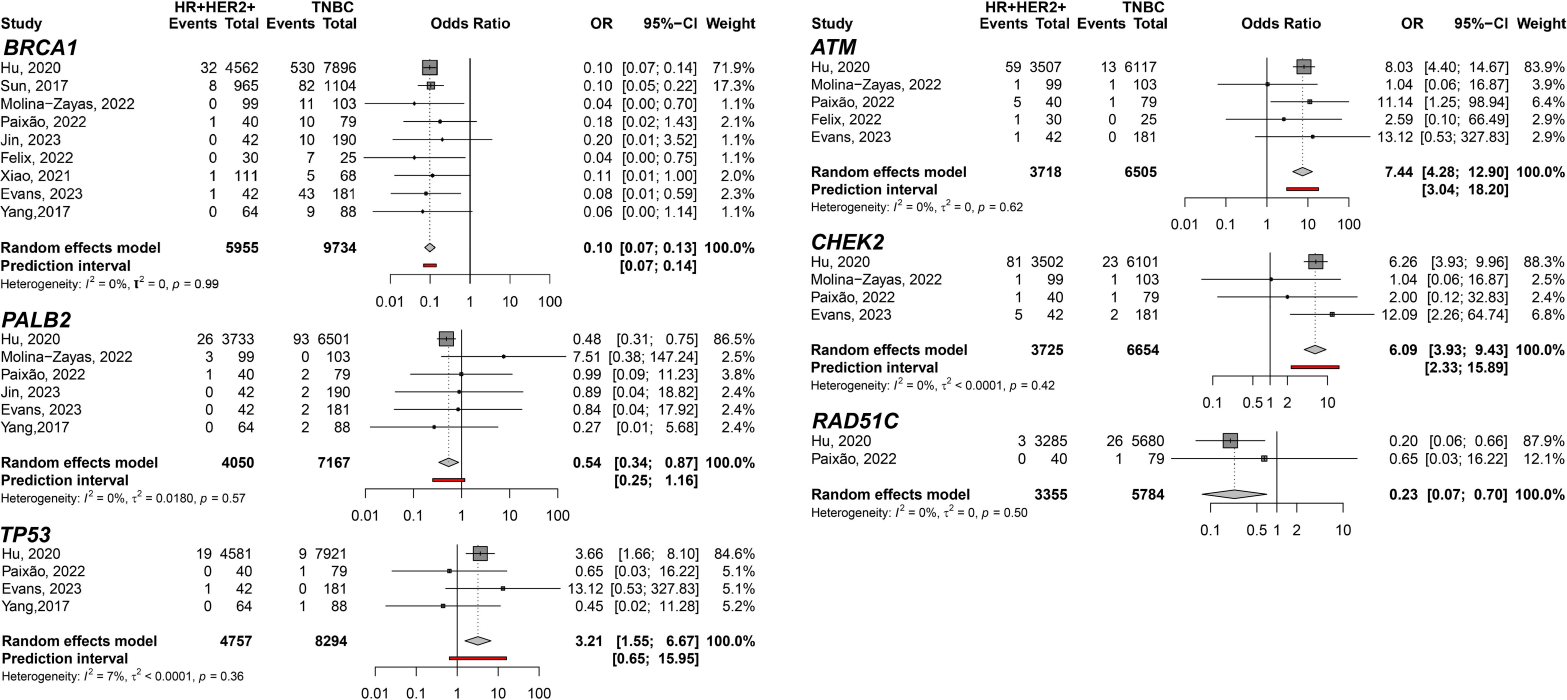
Forest plots showing the meta-analysis for the genes *BRCA1*, *PALB2*, *TP53*, *ATM*, *CHEK2*, and *RAD51C* related to the predisposition for HR+HER2+ (cases) breast cancer subtype to TNBC (controls).

**Figure 5:**
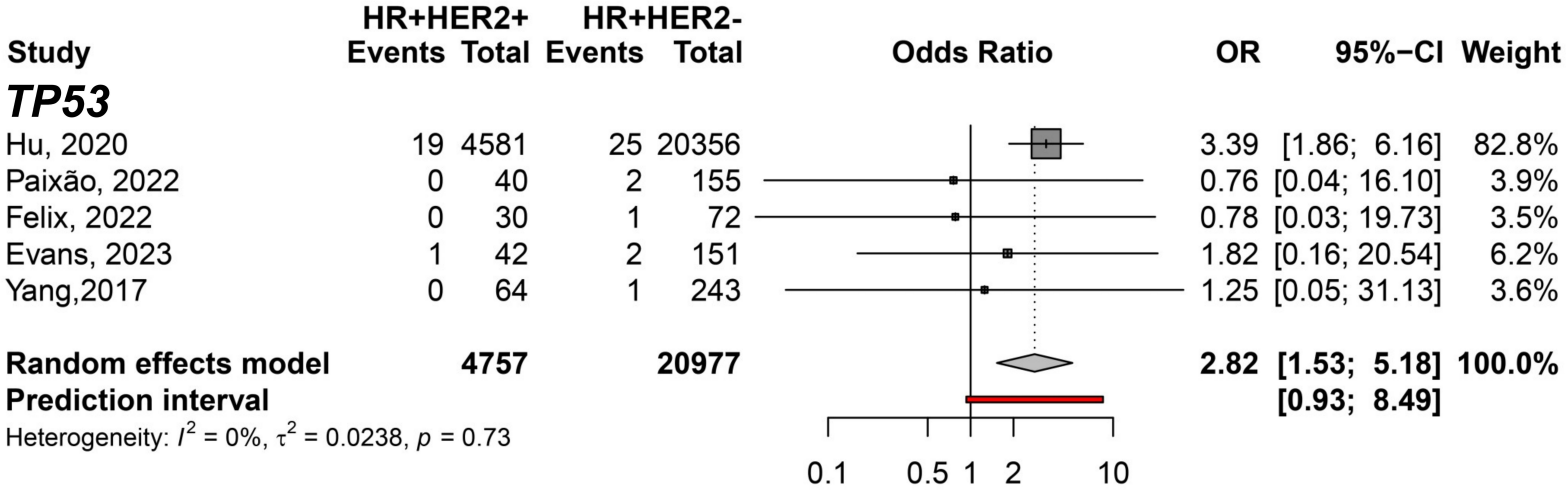
Forest plot showing the predisposing of HR+HER2+ (cases) breast cancer subtype compared to HR+HER2- (controls) for variant carriers on *TP53* gene.

In addition, the HR+HER2+ subtype compared to TNBC showed TP53 (pOR: 3.21; 95% CI 1.55–6.67; n=13,051); ATM (pOR: 7.44; 95% CI: 4.28–12.90, n=10,223); and CHEK2 (pOR: 6.09; 95% CI 3.93–9.43, n=10,379) predisposing to HR+HER2+. *BRCA1* (pOR: 0.10; 95% CI 0.07–0.13; n=12,917), *PALB2* (pOR: 0.54; 95% CI 0.34–0.87; n=11,217), and *RAD51C* (pOR: 0.23; 95% CI 0.07–0.70; n=9,139) (**Figure 4**). Germline variant frequencies for HR+HER2+ compared to HR+HER2- subtypes showed only the *TP53* germline variants differed significantly between HR+HER2+ and HR+HER2- BC patients, with a pOR of 2.82 (95% CI 1.53–5.18, n=25,734), predisposing to HR+HER2+ (**Figure 5**).

**Figure 6:**
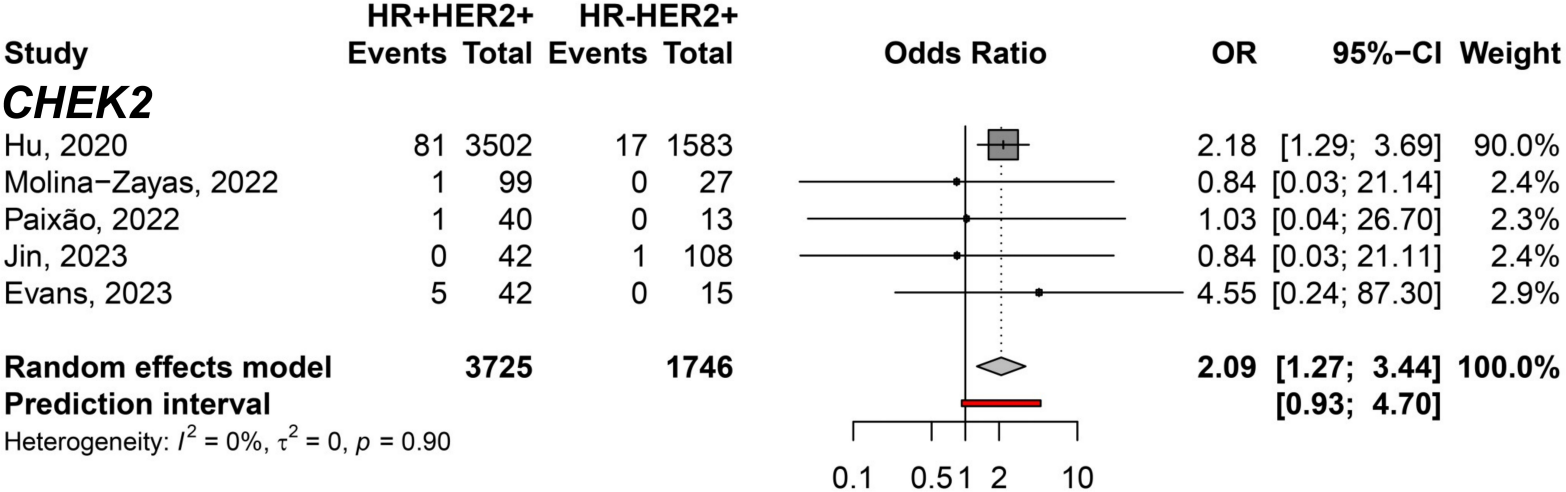
Forest plot showing the predisposition to HR+HER2+ (cases) breast cancer subtype compared to HR-HER2+ (controls) for the *CHEK2* gene.

**Figure 7:**
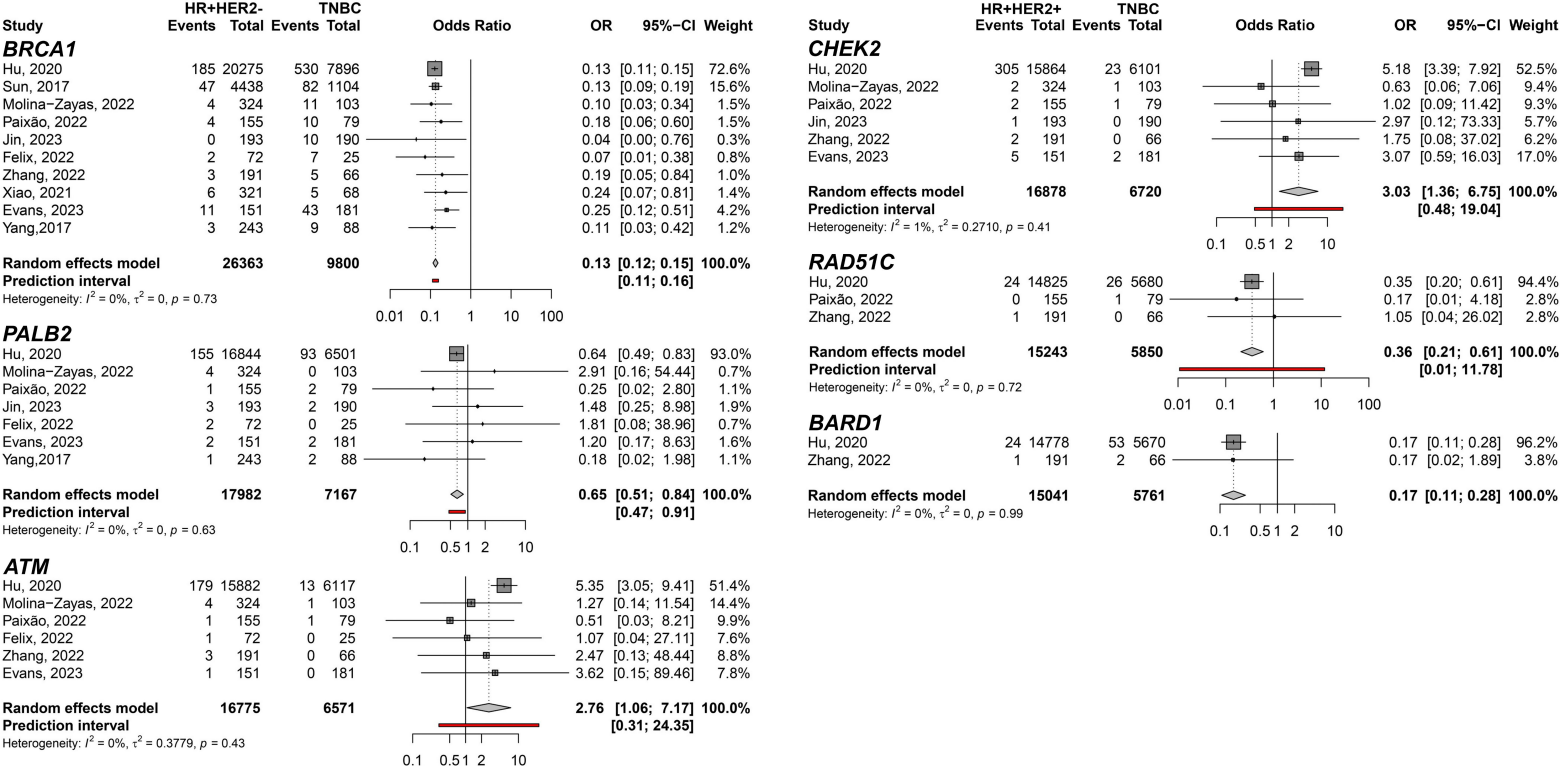
Forest plots of the comparison of HR+HER2- (cases) breast cancer subtype predisposition related to TNBC (controls) for the genes *BRCA1*, *PALB2*, *ATM*, *CHEK2*, *RAD51C*, and *BARD1*.

To filter out genes not directly associated with HER2 expression, we conducted a comparison exclusively within the groups exhibiting HER2 overexpression. The meta-analysis between HR+HER2+ and HR-HER2+ BC subtypes only showed significant differences in the *CHEK2* germline variant carriers predisposing to HR+HER2+ (pOR: 2.09; 95% CI 1.27–3.44, n=5,471) (**Figure 6**). Moreover, we identified germline variants that show consistent differences between HER2- groups to discover the less likely variants directly affecting HER2 and could be considered independent of HER2 expression when comparing HR+HER2- and TNBC groups. Six genes with germline variants were identified that differ in odds between both groups (**Figure 7**). *BRCA1* (pOR: 0.13; 95% CI: 0.12–0.15; n=36,163), *PALB2* (pOR: 0.65; 95% CI: 0.51–0.84; 25,149), *RAD51C* (pOR: 0.36; 95% CI: 0.21–0.61; n=21,093), and *BARD1* (pOR: 0.17; 95% CI: 0.11–0.28; n=20,802) were found predisposing to TNBC, while *ATM* (pOR: 2.76; 95% CI: 1.06–7.17; n=23,346) and *CHEK2* (pOR: 3.03; 95% CI: 1.36–6.75; n=23,598), predispose to HR+HER2.

#### 3.3.1 Shared characteristics across different comparisons

**Figure 8:**
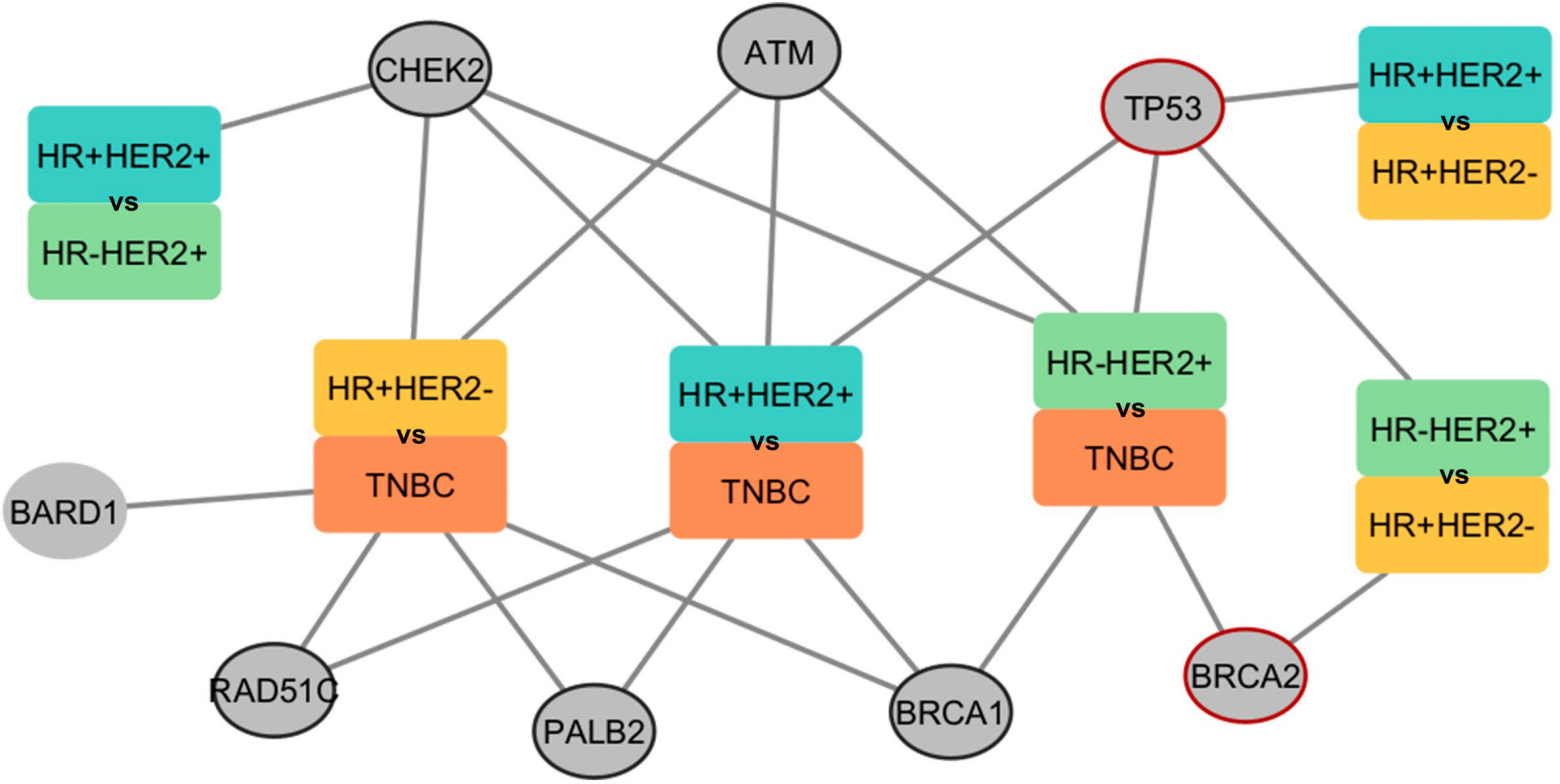
Network presenting the comparisons performed in the meta-analysis and the respective genes found predisposing to the BC subtype. The rectangles represent groups by subtypes of BC. Each color within the rectangle represents a subtype. Circles represent genes found in the associations. Circled circles represent genes found in any association comparing HER2+ with HER2-. Red-circled circles represent genes found exclusively in HER2+ with HER2- associations. The edges connect the gene and the subtype in which a higher predisposition was found.

In order to identify germline variants that could differently predispose BC subtypes, we compared the shared genes carrying germline variants between the comparisons. Germline variants of *TP53* proved to be exclusive when comparing HER2+ and HER2- subtypes (**Figure 8**). Also, germline variants within the *BRCA2* gene were found to predispose TNBC only when compared to HER2+ subtypes. Genes whose comparisons were not significant in the meta-analysis are described in **Supplementary Table 6**.

## 4 Discussion

It is known that approximately 10% of BC cases result from hereditary causes (33). Genetic testing has been widely implemented in BC care to determine hereditary cancer syndromes and personalized medicine (34). Here, we investigated the presence of rare germline variants in a specific group of genes with the aim of determining their possible association with HER2+ BC. We conducted a thorough systematic review and meta-analysis and compared the prevalence of these variants in subtypes characterized by HER2 overexpression with other well-defined BC subtypes. Of the 36 studies included in our analysis, 11 provided data on the distribution of germline variants in different clinically relevant BC subtypes, allowing comparative assessments. Our meta-analysis revealed significant differences in the occurrence of germline variants between BC groups for eight genes: *BRCA1, BRCA2, TP53, ATM, CHEK2, PALB2, RAD51C*, and *BARD1.*

In our analysis, we identified genes with germline variants that predispose to HER2 overexpressing subtypes: *TP53*, *ATM*, and *CHEK2*. Of these genes, germline variants on *TP53* gene were found to be exclusive when comparing HER2+ and HER2- subtypes, supporting a link between germline variants in this gene and increased expression of the HER2 protein. In fact, the proportion of HER2+ BC is increased in patients with LFS (35–38). Breast tumors in *TP53* germline variant carriers are usually high-grade, HER2+, and HR+ with a dense sclerotic tumor stroma (37).

The *ATM* germline variants were also significantly associated with subtypes overexpressing HER2 and/or HR, which is consistent with previous studies describing intermediate- to high-grade, HR+ disease and potentially higher rates of HER2 positivity and lymph node involvement associated with *ATM* germline variants (39, 40), as well as reduced recurrence time in invasive HER2+ BC patients (39). *ATM* truncating and missense variants are linked to an increased risk of estrogen receptor-positive (ER+) BC rather than ER- disease (41).

The *CHEK2* germline variants were found associated with both HR+ and HER2+ status. The highest chance was found when comparing the HR+HER2+ subtype with TNBC. A study investigating *CHEK2* germline variants identified an association of the immunophenotypic molecular subtypes of breast cancer with the type of the genetic variant. *CHEK2*-truncating variants were found to increase 6-fold the risk of luminal B (ER+ and/or PR+, HER2+) subtype (42). Moreover, other studies investigating the relation between *CHEK2* germline pathogenic variants and receptor status do not show concordance. Variants in the *CHEK2* gene were found to be associated with negative or positive status of HR and HER2 (43–45).

Remarkably, the TP53, ATM, and CHK2 proteins work together to control the cell cycle. When DNA damage occurs, ATM phosphorylates CHK2, triggering its activation. This in turn initiates a cascade of events that leads to the phosphorylation of various downstream substrates, including p53, which arrests the cell cycle and regulates apoptosis, and BRCA1, which modulates DNA repair (46). Interestingly, HER2 blockade indirectly affects downstream signaling pathways such as AKT, which could have secondary effects on CHK2 activity. In this sense, an effective treatment strategy targeting HER2 blockade would lead to inhibition of the AKT signaling pathway, thereby abrogating its inhibition on CHK2. This disruption would facilitate CHK2 function and enable p53-mediated repair of DNA damage. However, mutations in CHK2, p53, or ATM could impair this response by preventing CHK2 activity directly or by inhibiting CHK2 phosphorylation by ATM (47).

The present study confirms that *BRCA1* germline variants are consistently associated with a higher susceptibility to TNBC, with a lifetime risk of between 50 and 85% (48–50). In addition, *BRCA2* germline variants show an increased predisposition for HER2- groups (48, 49). Furthermore, *PALB2* germline variant carriers were found to have a higher predisposition to develop TNBC. In fact, BC in *PALB2* germline variant carriers has similar phenotypic characteristics to our results: 40% are TNBC, and 93% are HER2- (51). Moreover, germline variants on *BARD1* and *RAD51C* genes were found to predispose to TNBC. Interestingly, the BARD1 protein interacts with BRCA1 (BRCA1-BARD1) forming a tumor suppressor complex, which is an E3 ubiquitin ligase necessary for DNA double-strand breaks repair by RAD51-mediated homologous recombination (52). Identifying the three genes predisposing to TNBC may suggest a role of this pathway in the BC subtype development.

This study has some limitations. Smaller studies reporting lower OR for rare germline variants in individuals with BC are underrepresented in the scientific literature, possibly indicating a potential bias toward publishing studies with stronger or more significant findings. To address this bias, this work included only studies that perform multi-genic analysis. In addition, some publications focused only on a limited number of variants within their cohorts. Despite our efforts to ask authors for additional data for subgroup analyses, the meta-analyzed data were ultimately limited compared to larger cohorts. A risk of bias assessment revealed that confounding factors were identified in several studies, with no methods provided to address them. However, given the nature of the data we used for the meta-analysis, these confounders would not directly interfere with our analyses.

Finally, this study strengthens our understanding of the gene-specific risks associated with HER2 overexpression and provides crucial insights to identify genes that need to be tested in the context of HER2+ BC. Such findings could improve the management of hereditary BC by guiding the exploration of genes and prognosis of the disease through the correlation of gene-subtype BC. To our knowledge, this is the first meta-analysis to investigate genetic variants associated with HER2+ BC, through comparisons between BC subtypes. The meta-analysis results and literature review findings point to the importance of a closer examination of *TP53* germline variants in relation to breast tumors with overexpressed HER2. Also, *CHEK2* and *ATM* germline variants predisposed to HER2+ only when compared to TNBC and could be involved with HER2 overexpression more intricately. Furthermore, variants in *BRCA1* may confer a higher risk for the development of TNBC in comparison to other subtypes. This association was already suggested in the literature and the meta-analysis was able to confirm the evidence. Moreover, *BRCA2* germline variants exhibited a greater predisposition to HER2- when compared to HER2 overexpressing groups, implying a possible involvement of this gene in the HER2 expression.

## Data availability statement

The original contributions presented in the study are included in the article/**Supplementary Material**. Further inquiries can be directed to the corresponding author.

## Author contributions

AB: Conceptualization, Data curation, Formal analysis, Investigation, Methodology, Writing – original draft. NC: Conceptualization, Data curation, Formal analysis, Investigation, Methodology, Writing – original draft. LP: Data curation, Formal analysis, Investigation, Methodology, Writing – original draft. GC: Data curation, Formal analysis, Investigation, Writing – review & editing. JS: Investigation, Methodology, Writing – review & editing. MB: Conceptualization, Data curation, Formal analysis, Investigation, Methodology, Project administration, Writing – review & editing. CB: Funding acquisition, Validation, Writing – review & editing. MR: Funding acquisition, Validation, Writing – review & editing. GM: Conceptualization, Funding acquisition, Methodology, Supervision, Writing – review & editing. DR: Conceptualization, Funding acquisition, Methodology, Supervision, Writing – review & editing.
