# Supplementary material for: Germline rare variants in HER2-positive breast cancer predisposition: a systematic review and meta-analysis
Source: Front Oncol. 2024 Jun 24;14:1395970. doi: 10.3389/fonc.2024.1395970 (PMC11228612; doi:10.3389/fonc.2024.1395970)
Supplement: Supplementary file 1 [file DataSheet_1.pdf]

Supplementary Material

Germline rare variants in HER2-positive breast cancer predisposition: a systematic review and meta-analysis

Angelica Cerveira de Baumont, PhD[1]†*, Nathan Araujo Cadore, MSc[1,2]†, Luana Giongo Pedrotti, MSc[1], Giovana Dallaio Curzel[1], Jaqueline Bohrer Schuch, PhD[1], Marina Bessel, PhD[1], Cláudia Bordignon, MD[1], Mahira Lopes Rosa, MD[1], Gabriel de Souza Macedo, PhD[1], Daniela Dornelles Rosa, PhD[1,3]

[1]Hospital Moinhos de Vento, Porto Alegre, Brazil.
[2]Programa de Pós-Graduação em Genética e Biologia Molecular, Universidade Federal do Rio Grande do Sul, Porto Alegre, Brazil.
[3]Programa de Pós-Graduação em Ciências Médicas, Universidade Federal do Rio Grande do Sul, Porto Alegre, Brazil.

*** Correspondence:**
Angelica Cerveira de Baumont
angelica.baumont@hmv.org.br

1 Supplementary Methods

Search strategy

PubMed: (using MESH terms)

Breast cancer OR breast neoplasia OR breast carcinoma OR mammary cancer OR "Breast Neoplasms"[Mesh]

AND

HER2 OR ERBB2 OR Human Epidermal growth factor Receptor-type 2 OR CD340 OR Erb-B2 Receptor Tyrosine Kinase 2 OR "Receptor, ErbB-2"[Mesh] OR "Genes, erbB-2"[Mesh]

AND

"Whole Genome Sequencing"[Mesh] OR "Exome Sequencing"[Mesh] OR "High-Throughput Nucleotide Sequencing"[Mesh] OR genetic panel OR "Microarray Analysis"[Mesh] OR "Genome-Wide Association Study"[Mesh]

Scopus:

'Breast cancer' OR 'breast neoplasia' OR 'breast carcinoma' OR 'mammary cancer' OR 'Breast Neoplasms'

Supplementary Material

AND

HER2 OR ERBB2 OR ‘Human Epidermal growth factor Receptor-type 2’ OR CD340 OR ‘Erb-B2 Receptor Tyrosine Kinase 2’

AND

‘whole-exome sequencing’ OR ‘whole-genome sequencing’ OR ‘genetic panel’ OR 'high throughput sequencing'

EMBASE: (using EMTREE terms)

'breast cancer'/exp OR 'breast carcinoma'/exp OR 'breast tumor'/exp

AND

HER2 OR 'epidermal growth factor receptor 2'/exp OR 'erbb2 protein human'/exp OR 'erb b2 receptor tyrosine kinase 2'/exp

AND

'whole genome sequencing'/exp OR 'whole exome sequencing'/exp OR 'microarray analysis'/exp OR 'high throughput sequencing'/exp OR 'genome-wide association study'/exp

2 Supplementary Tables

Supplementary Table 1. PRISMA 2020 Checklist.

Section and Topic	Item #	Checklist item	Location where item is reported
**TITLE**			
Title	1	Identify the report as a systematic review.	p. 1
**ABSTRACT**			
Abstract	2	See the PRISMA 2020 for Abstracts checklist.	See bellow
**INTRODUCTION**			
Rationale	3	Describe the rationale for the review in the context of existing knowledge.	p. 2-3
Objectives	4	Provide an explicit statement of the objective(s) or question(s) the review addresses.	p. 3
**METHODS**			
Eligibility criteria	5	Specify the inclusion and exclusion criteria for the review and how studies were grouped for the syntheses.	p. 3
Information sources	6	Specify all databases, registers, websites, organisations, reference lists and other sources searched or consulted to identify studies. Specify the date when each source was last searched or consulted.	p. 3
Search strategy	7	Present the full search strategies for all databases, registers and websites, including any filters and limits used.	Supplementary Methods
Selection process	8	Specify the methods used to decide whether a study met the inclusion criteria of the review, including how many reviewers screened each record and each report retrieved, whether they worked independently, and if applicable, details of automation tools used in the process.	p. 3
Data collection process	9	Specify the methods used to collect data from reports, including how many reviewers collected data from each report, whether they worked independently, any processes for obtaining or confirming data from study investigators, and if applicable, details of automation tools used in the process.	p. 3-4
Data items	10a	List and define all outcomes for which data were sought. Specify whether all results that were compatible	p. 3-4

Section and Topic	Item #	Checklist item	Location where item is reported
		with each outcome domain in each study were sought (e.g. for all measures, time points, analyses), and if not, the methods used to decide which results to collect.	
	10b	List and define all other variables for which data were sought (e.g. participant and intervention characteristics, funding sources). Describe any assumptions made about any missing or unclear information.	p. 3-4
Study risk of bias assessment	11	Specify the methods used to assess risk of bias in the included studies, including details of the tool(s) used, how many reviewers assessed each study and whether they worked independently, and if applicable, details of automation tools used in the process.	p. 4
Effect measures	12	Specify for each outcome the effect measure(s) (e.g. risk ratio, mean difference) used in the synthesis or presentation of results.	p. 4
Synthesis methods	13a	Describe the processes used to decide which studies were eligible for each synthesis (e.g. tabulating the study intervention characteristics and comparing against the planned groups for each synthesis (item #5)).	p. 4-6
	13b	Describe any methods required to prepare the data for presentation or synthesis, such as handling of missing summary statistics, or data conversions.	p. 3-4
	13c	Describe any methods used to tabulate or visually display results of individual studies and syntheses.	p. 4
	13d	Describe any methods used to synthesize results and provide a rationale for the choice(s). If meta-analysis was performed, describe the model(s), method(s) to identify the presence and extent of statistical heterogeneity, and software package(s) used.	p. 4
	13e	Describe any methods used to explore possible causes of heterogeneity among study results (e.g. subgroup analysis, meta-regression).	p. 4
	13f	Describe any sensitivity analyses conducted to assess robustness of the synthesized results.	Not applicable
Reporting bias assessment	14	Describe any methods used to assess risk of bias due to missing results in a synthesis (arising from reporting biases).	p. 4
Certainty assessment	15	Describe any methods used to assess certainty (or confidence) in the body of evidence for an outcome.	p. 4
**RESULTS**			
Study selection	16a	Describe the results of the search and selection process, from the number of records identified in the search to the number of studies included in the review, ideally using a flow diagram.	p. 4-5; Fig. 1
	16b	Cite studies that might appear to meet the inclusion criteria, but which were excluded, and explain why they were excluded.	p. 4-5; Supplementary table 4
Study characteristics	17	Cite each included study and present its characteristics.	Supplementary tables 3; 4 and 6

Section and Topic	Item #	Checklist item	Location where item is reported
Risk of bias in studies	18	Present assessments of risk of bias for each included study.	Supplementary figures 2-4
Results of individual studies	19	For all outcomes, present, for each study: (a) summary statistics for each group (where appropriate) and (b) an effect estimate and its precision (e.g. confidence/credible interval), ideally using structured tables or plots.	Figures 2-7; Supplementary figures 5-10; Supplementary table 6
Results of syntheses	20a	For each synthesis, briefly summarise the characteristics and risk of bias among contributing studies.	p. 5-6; Supplementary table 3-5; Supplementary figures 2-4
	20b	Present results of all statistical syntheses conducted. If meta-analysis was done, present for each the summary estimate and its precision (e.g. confidence/credible interval) and measures of statistical heterogeneity. If comparing groups, describe the direction of the effect.	p. 5-6; Figures 2-7; Supplementary figures 5-10; Supplementary table 4 and 6
	20c	Present results of all investigations of possible causes of heterogeneity among study results.	Figures 2-7; Supplementary figures 5-10
	20d	Present results of all sensitivity analyses conducted to assess the robustness of the synthesized results.	Not applicable
Reporting biases	21	Present assessments of risk of bias due to missing results (arising from reporting biases) for each synthesis assessed.	p. 5; Supplementary figures 2-4
Certainty of evidence	22	Present assessments of certainty (or confidence) in the body of evidence for each outcome assessed.	Figures 2-7; Supplementary figures 5-10; Supplementary table 6
**DISCUSSION**			
Discussion	23a	Provide a general interpretation of the results in the context of other evidence.	p. 6-8
	23b	Discuss any limitations of the evidence included in the review.	p. 7
	23c	Discuss any limitations of the review processes used.	p. 7
	23d	Discuss implications of the results for practice, policy, and future research.	p. 7-8
**OTHER INFORMATION**			
Registration and protocol	24a	Provide registration information for the review, including register name and registration number, or state that the review was not registered.	p. 3
	24b	Indicate where the review protocol can be accessed, or state that a protocol was not prepared.	p. 3
	24c	Describe and explain any amendments to information provided at registration or in the protocol.	Not applicable

Supplementary Material

Section and Topic	Item #	Checklist item	Location where item is reported
Support	25	Describe sources of financial or non-financial support for the review, and the role of the funders or sponsors in the review.	p. 12
Competing interests	26	Declare any competing interests of review authors.	p. 6
Availability of data, code and other materials	27	Report which of the following are publicly available and where they can be found: template data collection forms; data extracted from included studies; data used for all analyses; analytic code; any other materials used in the review.	Not applicable

From: Page MJ, McKenzie JE, Bossuyt PM, Boutron I, Hoffmann TC, Mulrow CD, et al. The PRISMA 2020 statement: an updated guideline for reporting systematic reviews. BMJ 2021;372:n71. doi: 10.1136/bmj.n71

For more information, visit: http://www.prisma-statement.org/

Supplementary Table 2. PRISMA 2020 for Abstracts Checklist.

Section and Topic	Item #	Checklist item	Reported (Yes/No)
**TITLE**			
Title	1	Identify the report as a systematic review.	Yes
**BACKGROUND**			
Objectives	2	Provide an explicit statement of the main objective(s) or question(s) the review addresses.	Yes
**METHODS**			
Eligibility criteria	3	Specify the inclusion and exclusion criteria for the review.	Yes
Information sources	4	Specify the information sources (e.g. databases, registers) used to identify studies and the date when each was last searched.	Yes
Risk of bias	5	Specify the methods used to assess risk of bias in the included studies.	Yes
Synthesis of results	6	Specify the methods used to present and synthesise results.	Yes
**RESULTS**			
Included studies	7	Give the total number of included studies and participants and summarise relevant characteristics of studies.	Yes
Synthesis of results	8	Present results for main outcomes, preferably indicating the number of included studies and participants for each. If meta-analysis was done, report the summary estimate and confidence/credible interval. If comparing groups, indicate the direction of the effect (i.e. which group is favoured).	Yes
**DISCUSSION**			
Limitations of evidence	9	Provide a brief summary of the limitations of the evidence included in the review (e.g. study risk of bias, inconsistency and imprecision).	Yes
Interpretation	10	Provide a general interpretation of the results and important implications.	Yes

Supplementary Material

Section and Topic	Item #	Checklist item	Reported (Yes/No)
**OTHER**			
Funding	11	Specify the primary source of funding for the review.	Yes
Registration	12	Provide the register name and registration number.	Yes

From: Page MJ, McKenzie JE, Bossuyt PM, Boutron I, Hoffmann TC, Mulrow CD, et al. The PRISMA 2020 statement: an updated guideline for reporting systematic reviews. BMJ 2021;372:n71. doi: 10.1136/bmj.n71

Supplementary Table 3: Studies included in the meta-analysis and the criteria applied for variant classification.

Study	Criteria
Hu et al. 2020	Germline truncating, consensus dinucleotide splice sites (+/−1 or 2), and any known pathogenic missense alterations assessed by a five-tier classification system as proposed by the Unclassified Genetic Variants Working Group of the International Agency for Research on Cancer (1).
Sun et al. 2017	Only protein-truncating variants were included. For splice-site, variants were included when verified by functional analyses from the literature. For synonymous, nonsynonymous, in-frame, and stop-loss variants, only variants classified as pathogenic or probably pathogenic by ClinVar (https://www.ncbi.nlm.nih.gov/clinvar/) were included. The variant data were further assessed and classified according to the American College of Medical Genetics and Genomics guidelines (2).
Molina-Zayas et al. 2022	Variants were classified using the American College of Medical Genetics and Genomics (2) guidelines and variants of uncertain significance (VUS) were classified as pathogenic by SIFT (3), PolyPhen-2 (4), and MutationTaster (5) tools.
Paixão et al. 2022	Variants were classified using the American College of Medical Genetics and Genomics guidelines (2).
Jin et al. 2023	Variants were classified using the American College of Medical Genetics and Genomics guidelines (2).
Felix et al. 2022	Variants loss-of-function or experimentally demonstrated to damage gene function. Interpretations of possible splice variants were based on *in silico* algorithms or experimentally.
Zhang et al. 2022	Variants were classified using the American College of Medical Genetics and Genomics guidelines (2).
Xiao et al. 2021	Variants were filtered based on allele frequency (< 0.1%), annotated with ANNOVAR (6), and SnpEff (7).
Evans et al. 2023	Variants were classified following the American College of Medical Genetics (2) and Cancer Variant Interpretation Group guidelines (8).
Yang et al. 2017	Pathogenic variants included known pathogenic alleles in ClinVar (https://www.ncbi.nlm.nih.gov/clinvar/) and unreported loss of function variants (frameshift and stop-gain) or variants that alter splice site. Variants of unknown significance included variants by ClinVar, non-synonymous that have never been reported, and rare synonymous or intronic variants that were predicted to be deleterious.
Nikitin et al. 2020	Pathogenic variants predicted by SIFT (3), PolyPhen-2 (4), MutationTaster (5), CADD (9), DANN (10), M-CAP (11), or REVEL (12) tools.

Supplementary Material

1. Plon SE, Eccles DM, Easton D, Foulkes WD, Genuardi M, Greenblatt MS, Hogervorst FB, Hoogerbrugge N, Spurdle AB, Tavtigian SV; IARC Unclassified Genetic Variants Working Group. Sequence variant classification and reporting: recommendations for improving the interpretation of cancer susceptibility genetic test results. Hum Mutat. 2008
2. Richards S, Aziz N, Bale S, Bick D, Das S, Gastier-Foster J, Grody WW, Hegde M, Lyon E, Spector E, Voelkerding K, Rehm HL; ACMG Laboratory Quality Assurance Committee. Standards and guidelines for the interpretation of sequence variants: a joint consensus recommendation of the American College of Medical Genetics and Genomics and the Association for Molecular Pathology. Genet Med. 2015 May;17(5):405-24. doi: 10.1038/gim.2015.30. Epub 2015 Mar 5. PMID: 25741868; PMCID: PMC4544753.
3. Sim NL, Kumar P, Hu J, Henikoff S, Schneider G, Ng PC. SIFT web server: predicting effects of amino acid substitutions on proteins. Nucleic Acids Res. 2012 Jul;40(Web Server issue):W452-7. doi: 10.1093/nar/gks539. Epub 2012 Jun 11. PMID: 22689647; PMCID: PMC3394338.
4. Adzhubei IA, Schmidt S, Peshkin L, Ramensky VE, Gerasimova A, Bork P, Kondrashov AS, Sunyaev SR. A method and server for predicting damaging missense mutations. Nat Methods. 2010 Apr;7(4):248-9. doi: 10.1038/nmeth0410-248. PMID: 20354512; PMCID: PMC2855889.
5. Schwarz JM, Rödelsperger C, Schuelke M, Seelow D. MutationTaster evaluates disease-causing potential of sequence alterations. Nat Methods. 2010 Aug;7(8):575-6. doi: 10.1038/nmeth0810-575. PMID: 20676075.
6. Wang K, Li M, Hakonarson H. ANNOVAR: functional annotation of genetic variants from high-throughput sequencing data. Nucleic Acids Res. 2010 Sep;38(16):e164. doi: 10.1093/nar/gkq603. PMID: 20601685; PMCID: PMC2938201.
7. Cingolani P, Platts A, Wang le L, Coon M, Nguyen T, Wang L, Land SJ, Lu X, Ruden DM. A program for annotating and predicting the effects of single nucleotide polymorphisms, SnpEff: SNPs in the genome of Drosophila melanogaster strain w1118; iso-2; iso-3. Fly (Austin). 2012 Apr-Jun;6(2):80-92. doi: 10.4161/fly.19695. PMID: 22728672; PMCID: PMC3679285.
8. Garrett A, Callaway A, Durkie M, Cubuk C, Alikian M, Burghel GJ, Robinson R, Izatt L, Talukdar S, Side L, Cranston T, Palmer-Smith S, Baralle D, Berry IR, Drummond J, Wallace AJ, Norbury G, Eccles DM, Ellard S, Lalloo F, Evans DG, Woodward E, Tischkowitz M, Hanson H, Turnbull C; CanVIG-UK. Cancer Variant Interpretation Group UK (CanVIG-UK): an exemplar national subspecialty multidisciplinary network. J Med Genet. 2020 Dec;57(12):829-834. doi: 10.1136/jmedgenet-2019-106759. Epub 2020 Mar 13. PMID: 32170000; PMCID: PMC7691806.
9. Kircher M, Witten DM, Jain P, O'Roak BJ, Cooper GM, Shendure J. A general framework for estimating the relative pathogenicity of human genetic variants. Nat Genet. 2014 Mar;46(3):310-5. doi: 10.1038/ng.2892. Epub 2014 Feb 2. PMID: 24487276; PMCID: PMC3992975.
10. Quang D, Chen Y, Xie X. DANN: a deep learning approach for annotating the pathogenicity of genetic variants. Bioinformatics. 2015 Mar 1;31(5):761-3. doi: 10.1093/bioinformatics/btu703. Epub 2014 Oct 22. PMID: 25338716; PMCID: PMC4341060.
11. Jagadeesh KA, Wenger AM, Berger MJ, Guturu H, Stenson PD, Cooper DN, Bernstein JA, Bejerano G. M-CAP eliminates a majority of variants of uncertain significance in clinical exomes at high sensitivity. Nat Genet. 2016 Dec;48(12):1581-1586. doi: 10.1038/ng.3703. Epub 2016 Oct 24. PMID: 27776117.
12. Ioannidis NM, Rothstein JH, Pejaver V, Middha S, McDonnell SK, Baheti S, Musolf A, Li Q, Holzinger E, Karyadi D, Cannon-Albright LA, Teerlink CC, Stanford JL, Isaacs WB, Xu J, Cooney KA, Lange EM, Schleutker J, Carpten JD, Powell IJ, Cussenot O, Cancel-Tassin G, Giles GG, MacInnis RJ, Maier C, Hsieh CL, Wiklund F, Catalona WJ, Foulkes WD, Mandal D, Eeles RA, Kote-Jarai Z, Bustamante CD, Schaid DJ, Hastie T, Ostrander EA, Bailey-Wilson JE, Radivojac P, Thibodeau SN, Whittemore AS, Sieh W. REVEL: An Ensemble Method for Predicting the Pathogenicity of Rare Missense Variants. Am J Hum Genet. 2016 Oct 6;99(4):877-885. doi: 10.1016/j.ajhg.2016.08.016. Epub 2016 Sep 22. PMID: 27666373; PMCID: PMC5065685.

Supplementary Table 4. Studies included in the data extraction.

doi	Title	Author	Year	Country	Ethnicity/nationality/ ancestry	Genomic assessment	Study design	Population	Genes with rare variants identified
10.1093/jnci/djaa023	The Contribution of Germline Predisposition Gene Mutations to Clinical Subtypes of Invasive Breast Cancer From a Clinical Genetic Testing Cohort	Hu et al.	2020	United States	Ashkenazi Jews (4.8%); Asian (4.8%); Black (8.1%); Hispanic (6.0%); Non-Hispanic (63.6%); Other/unknown (12.6%)	Targeted Panel (<100 genes)	Cross sectional	Female invasive breast cancer patients	*ATM; BARD1; BRCA1; BRCA2; BRIP1; CDH1; CHEK2; MSH6; NBN; NF1; PALB2; PTEN; RAD51C; RAD51D; TP53*
10.1158/1078-0432.CCR-16-3227	Germline Mutations in Cancer Susceptibility Genes in a Large Series of Unselected Breast Cancer Patients	Sun et al.	2017	China	Han Chinese (95.8%); Other (4.2%)	Targeted Panel (<100 genes)	Prospective cohort	Breast cancer patients	*BRCA1; BRCA2; PALB2; TP53; RAD51D; ATM; RECQL; CHEK2; BARD1; BLM; FANCC; PTEN; XRCC2; STK11; PMS1; MLH1; PMS2; POLE; MSH2; MSH6; EPCAM; POLD1; KIT; CTNNA1; ERCC2; VHL; MET; CDKN2A; BAP1; RB1; FLCN; PRF1; EZH2; RET*
10.1111/febs.13352	Detection of novel germline mutations for breast cancer in non-BRCA1/2 families.	Aloraifi et al.	2015	Ireland	Irish/ European ancestry	Large Panel (> 100 genes)	Case-control	Cases of familial breast cancer without BRCA1/2 mutations	*ABCC11; AKAP9; ANKLE1; ATM; AURKB; CASP5; CASP8; CCDC170; CCNB1; CCNE1; CD9; CDKN2B-AS1SPLICE_SITE_ACCEPTOR; CSMD3; CXCL6; CYP1B1; DSC3; ERBB2; ERCC2; ESR2; FANCA; FANCE; FANCI; FANCL; FANCM; FAT3; FLNB; FLNC; FOXM1; GABRA6; GHSR; IFI16; IGSF22; KDM5A; KDM5B; KDM6A; KIAA1919; LAMA3; LEPR;*

Supplementary Material

									MAML2; MAP3K1; MCAT; MLH1; MMP2; MRE11A; MSH2; MSH3; MSH6; MTHFD1; MTHFR; MUTYH; NBN; NOS3; NOTCH1; NQO1; OBSCN; PALB2; PLK1; PMS1; POR; RAD50; RAD51B; SBNO1; SFN; SLIT2; SPTAN1; SYNE2; TP53; TSC2; TTN; WRN; XRCC3; ZNF217; ZNF582
10.1093/annonc/mdv592	Genetic testing in a cohort of young patients with HER2-amplified breast cancer.	Eccles et al.	2016	United Kingdom	Asian (3%); Caucasian/ White (93%); Black (3%); Other (1%); Missing/unknown (1%)	Targeted Panel (<100 genes)	Cross sectional	HER2-amplified primary breast cancer patients	*BRCA1; BRCA2; TP53; ATM; BLM; BRIP1; CHEK2; NBN; PALB2*
10.1186/s13058-020-01273-y	Tumor sequencing is useful to refine the analysis of germline variants in unexplained high-risk breast cancer families.	Marcke et al.	2020	Belgium	European	Whole-exome sequencing (WES)	Cross sectional	Breast and/or ovarian cancer patients without BRCA1/2, TP53, and CHEK2 mutations	*ABCD4; ABL1; ABL2; AKAP9; ALK; ANKLE1; APC; APEX1; ATAD5; ATM; ATR; ATXN7; AURKA; AXIN2; BABAM1; BAP1; BARD1; BLM; BRCA2; BRIP1; CCDC88C; CCNE1; CDH1; CDH11; CDKN2A; CDYL2; CEP192; CREBBP; CRISPLD2; CTLA4; CYLD; DICER1; EDC4; EPCAM; ERBB2; ERBB4; ERCC2; ERCC3; ERCC4; ERCC6; ERCC8; EXO1; FANCC; FANCD2; FANCE; FANCF; FANCI; FANCM; FTO; GATA2; IGF2R; JAK2; JAKMIP3; KIT; LGR6; LIG1; LIG4; MAP3K1; MBD4; MDM2; MEN1; MET; MITF; MKL1; MLH1; MLH3; MPG; MRE11A; MSH2; MSH3; MSH5; MSH6; MTHFR; MUTYH; MYCN; MYO1E; MYOF; NBN; NEK10; NF1; NF2; NOTCH1; NOTCH2; NTHL1; NUMA1; OGG1; PALB2; PARP1; PBRM1; PDE4A; PDGFRB; PEX14; PFAS; PML; PMS1;*

									PMS2; POLB; POLD3; POLE; PTPN13; PTPN5; RABEP1; RAD1; RAD50; RAD51B; RAD51C; RAD52; RALY; RBBP8; RECQL; RECQL5; RELN; RFC1; RIN3; ROS1; SDHD; SMARCA4; SMG1; SMG7; SMUG1; SOS1; SUFU; TCOF1; TERT; TET2; TGFB2; TGFBR1; TGFBR3; TOX3; TP53; TRIM31; TRIM33; TSC1; TSC2; UIMC1; WRN; XPA; XPC; XRCC3
10.3390/genes13081362	Multi-Gene Mutation Profiling by Targeted Next-Generation Sequencing in Premenopausal Breast Cancer.	Zografos et al.	2022	Greece	Greek descent	Targeted Panel (<100 genes)	Cross sectional	Premenopausal breast cancer patients	*BRCA1; BRCA2; CHEK2; TP53; RAD51C; RAD51D; BRIP1; MUTYH; PMS2; ATM*
10.18632/oncotarget.7027	Multiple gene sequencing for risk assessment in patients with early-onset or familial breast cancer.	Lin et al.	2016	China	Han Chinese	Targeted Panel (<100 genes)	Cross sectional	Breast cancer patients with an early-onset or a significant family history	*ATM; BRCA1; BRCA2; BRIP1; FANCI; MSH2; MUTYH; RAD50; RAD51C; TP53*
10.1007/s10549-015-3545-6	Exome sequencing reveals frequent deleterious germline variants in cancer susceptibility genes in women with invasive breast cancer undergoing neoadjuvant chemotherapy.	Ellingson et al.	2015	United States	American Indian or Alaska Native (0.8%); Asian (2.4%); Black or African American (5.6%); White (88.7%); Unknown (2.4%)	Large Panel (> 100 genes)	Cross sectional	Stage I–III breast cancer patients recommended for neoadjuvant chemotherapy	*BRCA1; BRCA2; FH; ATM; BLM; CHEK2; FANCA; FANCI; FANCL; FANCM; MLH3; MUTYH; PALB2; WRN*
10.1007/s00438-022-01891-5	Identification of hereditary breast and ovarian cancer germline variants in Granada (Spain): NGS perspective.	Molina-Zayas et al.	2022	Spain	Spanish	Targeted Panel (<100 genes)	Retrospective cohort	High-risk patients for hereditary breast and/or ovarian cancer	*BRCA1; BRCA2; ATM; PALB2; CHEK2; MSH6; RAD51C; TP53*
10.1002/ijc.31601	Germline mutations in 40 cancer susceptibility genes among Chinese patients with	Li et al.	2019	China	Han Chinese (96.5%); Other (4.5%)	Targeted Panel (<100 genes)	Cross sectional	High-risk patients for	*BRCA1; BRCA2; TP53; CDH1; PALB2; ATM; CHEK2; BARD1; BRIP1; RAD50; MSH2; PMS2;*

	high hereditary risk breast cancer.							hereditary breast cancer	*MLH1; PMS1; NQO2; PPM1D; ERBB2*
10.1186/s13073-021-00978-9	Germline breast cancer susceptibility genes, tumor characteristics, and survival.	Ho et al.	2021	Singapore, Malaysian and Korean	Chinese (71%); Indian 707 (8%); Korean (6%); Malay(14%); Other (1%)	Targeted Panel (<100 genes)	Cohort	Breast cancer patients of Asian descent	*ABRAXIS1; AKT1; ATM; BABAM2; BARD1; BRCA1; BRCA2; BRIP1; CDH1; CHEK2; EPCAM; FANCC; FANCM; GEN1; MEN1; MRE11A; MSH6; MUTYH; NBN; NF1; PALB2; PIK3CA; PMS2; PTEN; RAD50; RAD51C; RAD51D; RECQL; RINT1; STK11; TP53; XRCC2*
10.1001/jamaoncol.2021.6744	Pathology of Tumors Associated With Pathogenic Germline Variants in 9 Breast Cancer Susceptibility Genes.	Mavaddat et al.	2022	Europe and East Asia	European or East Asian ethnicity	Targeted Panel (<100 genes)	Case-control	Breast cancer patients and unaffected control participants	*ATM; BARD1; BRCA1; BRCA2; CHEK2; PALB2; RAD51C; RAD51D; TP53*
10.1002/cam4.1376	Gene panel testing of 5589 BRCA1/2-negative index patients with breast cancer in a routine diagnostic setting: results of the German Consortium for Hereditary Breast and Ovarian Cancer.	Hauke et al.	2018	Germany	German/ European descent	Targeted Panel (<100 genes)	Case-control	Brast cancer patients negative for pathogenic BRCA1/2 mutations and control participants	*ATM; CDH1; CHEK2; NBN; PALB2; RAD51C; RAD51D; TP53*
10.3389/fonc.2022.976959	Characterization of genetic predisposition to molecular subtypes of breast cancer in Brazilian patients.	Paixão et al.	2022	Brazil	Predominant African Ancestry (3.7%); Predominant Asian/Native American Ancestry (5.0%); Predominant European Ancestry (57.0%)	Targeted Panel (<100 genes)	Cross sectional	Breast cancer unrelated patients	*BRCA1; BRCA2; TP53; PALB2; ATM; CHEK2; RAD51C; MUTYH; SBDS; FANCI; HNF1A; PFR1; RECQL4; BLM; BRIP1; FANCA; FANCD2; FANCE; FANCL; FANCM; FH; PHOX2B; PMS2; SLX4*
10.18632/oncotarget.27704	Sequencing for an interdisciplinary molecular tumor board in patients with advanced breast cancer: experiences from a case series.	Walter et al.	2020	Germany	European	Large Panel (> 100 genes)	Retrospective cohort	Metastatic breast cancer patients	*BRCA1; PALB2; TP53; MLH1; MSH3*

10.1186/s12967-023-04076-9	Analysis of clinical features, genomic landscapes and survival outcomes in HER2-low breast cancer	Jin et al.	2023	China	Chinese	Large Panel (> 100 genes)	Retrospective cohort	Metastatic breast cancer patients	*BRCA1; BRCA2; PALB2; CHEK2; VEGFA; MUTYH; WRN*
10.4274/ejbh.galenos.2022.2022-7-2	Genetic, Surgical and Oncological Approach to Breast Cancer, with BRCA1, BRCA2, CDH1, PALB2, PTEN and TP53 Variants	Subaşıoğlu et al.	2023	Turkey	Turkish	Targeted Panel (<100 genes)	Cross sectional	Patients with operated breast cancer	*BRCA1; BRCA2; CDH1; PALB2; PTEN; TP53*
10.3390/ijms231911266	Germline Variants in 32 Cancer-Related Genes among 700 Chinese Breast Cancer Patients by Next-Generation Sequencing: A Clinic-Based, Observational Study	Yang et al.	2022	China	Han Chinese (99.4%); Mongolian (0.3%); Uyghur (0.3%)	Targeted Panel (<100 genes)	Cross sectional	Breast cancer patients	*TP53; BRCA2; BRCA1; PALB2; NBN; MRE11A; BARD1; CHEK1; CHEK2; ATR; BRIP1; FANCL; RAD51C; RAD51D; RAD54L*
10.1007/s10549-022-06560-0	Mutational spectrum of breast cancer susceptibility genes among women ascertained in a cancer risk clinic in Northeast Brazil	Felix et al.	2022	Brazil	Cases: African-descended (67%); White (26%); Other (6.9%); Unaffected: African-descended (90.8%); White (8.4%); Other (0.8%)	Targeted Panel (<100 genes)	Case-control	Breast cancer patients from Bahia/ BR	*BRCA1; BRCA2; PALB2; ATM; BRIP1; BARD1; FAM175A; FANCM; NBN; SLX4; TP53; RAD51C*
10.1158/1078-0432.CCR-21-2572	Somatic and Germline Genomic Alterations in Very Young Women with Breast Cancer	Waks et al.	2022	United States/ Canada	Race: Asian (7.6%); Caucasian (85.9%); Multi-racial (3.3%); Ethnicity: Hispanic or Latin (6.5%), Non–Hispanic or Latin (88%); Unknown (5.4%)	Whole-exome sequencing (WES)	Prospective Cohort	Nonmetastatic breast cancer patients ≤35 years old	*BRCA1; BRCA2; PALB2; BUB1B; TP53; PRSS1; GJB2; COL7A1*

Supplementary Material

10.3389/fonc.2022.745796	Germline Mutational Landscape in Chinese Patients With Advanced Breast Cancer	Zhang et al.	2022	China	Chinese	Large Panel (> 100 genes)	Retrospective cohort	Advanced breast cancer patients	*ABRAXAS1; ATM; ATR; BARD1; BRCA1; BRCA2; BRIP1; CHEK2; ERCC2; FANCC; FANCD2; FANCE; FANCG; FANCL; FANCM; MRE11; NBN; PMS2; RAD50; RAD51C; RAD51D; STK11; TP53; XRCC1*
10.3389/fonc.2021.797505	The Mutational Landscape of Early-Onset Breast Cancer: A Next-Generation Sequencing Analysis	Andrikopoulou et al.	2022	Greece	Greek	Targeted Panel (<100 genes)	Cross sectional	Young (<40 years) and older breast cancer patients	*BRCA1; CHEK2; ATM; BRCA2; APC; TP53*
10.3390/cancers13164154	Gene panel testing for breast cancer reveals differential effect of prior BRCA1/2 probability	Evans et al.	2021	United Kingdom	UK population	Targeted Panel (<100 genes)	Case-control	Female breast cancer patients	*BRCA1; BRCA2; ATM; CHEK2; TP53; PALB2; NBN; CDH1; RAD51C; RAD51D; BRIP1; BARD1; PTEN; RECQL*
10.3389/fgene.2021.674094	Characteristics of Germline Non-BRCA Mutation Status of High-Risk Breast Cancer Patients in China and Correlation with High-Risk Factors and Multigene Testing Suggestions	Su et al.	2021	China	Chinese	Targeted Panel (<100 genes)	Retrospective cohort	High-risk breast cancer patients	*ATM; ATR; BARD1; BRCA1; BRCA2; BRIP1; CHEK2; ERCC3; FANCA; HOXB13; MLH1; MRE11; PALB2; PMS2; RAD51C; RAD51D; RAD54L; TP53*
10.3389/fonc.2021.618767	Characterization of Frequently Mutated Cancer Genes and Tumor Mutation Burden in Chinese Breast Cancer	Xiao et al.	2021	China	Chinese	Large Panel (> 100 genes)	Retrospective cohort	Early-stage breast cancer patients	*BRCA1; BRCA2; ATM; BARD1; BRIP1; CDH1; CHEK1; FANCA; FANCL; MUTYH; PALB2; PMS2; PTEN; RAD51C; SDHA; TP53*
10.1016/j.tranon.2020.100986	Determining homologous recombination deficiency scores with whole exome sequencing and their association with responses to neoadjuvant chemotherapy in breast cancer	Kim et al.	2021	Japan	Japanese	Whole-exome sequencing (WES)	Retrospective cohort	Stage II - IV breast cancer patients recommended for neoadjuvant chemotherapy	*BRCA1; BRCA2; RAD54B; PALB2*
10.3389/fonc.2020.568786	Mutational Landscape for Indian Hereditary Breast and Ovarian Cancer Cohort Suggests Need for Identifying	Kadri et al.	2020	India	Indian	Targeted Panel (<100 genes)	Cross sectional	Breast cancer women with a family history	*BRCA1; BRCA2; TP53; PALB2; BRIP1; ATM*

	Population Specific Genes and Biomarkers for Screening							of breast or ovary cancer	
10.1038/s10038-020-0729-7	Germline mutations of multiple breast cancer-related genes are differentially associated with triple-negative breast cancers and prognostic factors	Hata et al.	2020	China	Han Chinese	Targeted Panel (<100 genes)	Cross sectional	Breast cancer women	*BRCA2; BRCA1; PALB2; CHEK2; GEN1; BLM; RAD51D; RECQL; MRE11A; FANCM; BRIP1; MSH6; PMS2; MSH2; PMS1; MUTYH; TP53; PTEN; MET; NF1; AXIN1*
10.1136/jmg-2023-109196	Detection of pathogenic variants in breast cancer susceptibility genes in bilateral breast cancer.	Evans et al.	2023	United Kingdom	North West England	Targeted Panel (<100 genes)	Case-control	Bilateral breast cancer women	*BRCA1; BRCA2; TP53; CHEK2; BRIP1; PALB2; ATM; NBN; RAD51D*
10.1007/s10549-017-4356-8	Prevalence and spectrum of germline rare variants in BRCA1/2 and PALB2 among breast cancer cases in Sarawak, Malaysia	Yang et al.	2017	Malaysia	Chinese (46.3%), Malay (24.1%), Natives (27.2%), Others (2.4%)	Targeted Panel (<100 genes)	Prospective cohort	Invasive breast cancer patients	*BRCA1; BRCA2; PALB2; TP53*
10.1186/bcr3584	Evaluation of ultra-deep targeted sequencing for personalized breast cancer care	Harismendy et al.	2013	United States	California (USA)	Targeted Panel (<100 genes)	Cross sectional	Breast cancer patients	*BRCA1; BRCA2; DPYD; CFTR*
10.3389/fonc.2020.00666	Lynch Syndrome Germline Mutations in Breast Cancer: Next Generation Sequencing Case-Control Study of 1,263 Participants	Nikitin et al.	2020	Russia	Slavic, Tatar or Bashkir ethnicities	Targeted Panel (<100 genes)	Case-control	Hereditary and sporadic breast cancer patients and healthy controls	*MLH1; MSH2; MSH6; PMS2*

Supplementary Table 5. Modified Newcastle-Ottawa Quality Assessment Scale for Genetic Studies with awarded stars per category.

Study	Selection	Comparability	Outcome	Total
Hu, 2020	★★★☆	★★	☆☆	★★★★★
Sun, 2017	★★★☆	☆☆	☆☆	★★★☆☆
Lin, 2016	★★★☆	☆☆	☆☆	★★★☆☆
Molina-Zayas, 2022	★★★☆	☆☆	☆☆	★★★☆☆
Paixão, 2023	★★★☆	★★	☆☆	★★★★★
Jin, 2023	★★★☆	★★	★☆	★★★★★★
Felix, 2022	★★★☆	☆☆	☆☆	★★★☆☆
Xiao, 2021	★★★☆	☆☆	★☆	★★★★☆
Yang, 2017	★★★☆	★★	☆☆	★★★★★
Zhang, 2022	★★★☆	★★	★☆	★★★★★★
Evans, 2023	★★★☆	☆☆	☆☆	★★★☆☆

Supplementary Table 6. Genes that exhibited no significant variance when subjected to meta-analysis across different breast cancer subtypes.

Comparison	Gene	Number of studies	Authors	Total sample size in cases	Total sample size in controls	Odds Ratio (IC 95%)	Heterogeneity	p
1. HR-HER2+ *versus* TNBC	*MSH6*	2	Hu, 2020; Molina−Zayas, 2022	878	3336	1.75 (0.07; 43.90)	65%	0.09
	BRIP1	3	Hu, 2020; Felix, 2022; Evans, 2023	1530	5965	1.00 (0.09; 11.23)	57%	0.10
	PALB2	5	Hu, 2020; Paixão, 2022; Jin, 2023; Evans, 2023; Yang, 2017	1902	7167	0.50 (0.22; 1.11)	0%	0.62
	MUTYH	2	Paixão, 2022; Jin, 2023	121	269	2.40 (0.26; 22.24)	0%	0.50
	RAD51C	2	Hu, 2020; Paixão, 2022	1515	5784	0.66 (0.24; 1.79)	0%	0.49
2. HR-HER2+ *versus* HR+HER2-	*ATM*	5	Hu, 2020; Molina−Zayas, 2022; Paixão, 2022; Felix, 2022; Evans, 2023	1659	16584	1.02 (0.63; 1.64)	0%	0.87
	MSH6	2	Hu, 2020; Molina−Zayas, 2022	878	9470	3.36 (0.05; 248.43)	81%	0.02
	FANCM	2	Paixão, 2022; Felix, 2022	31	227	2.21 (0.22; 21.96)	0%	0.64
	RAD51D	2	Hu, 2020; Evans, 2023	1475	14752	1.65 (0.29; 9.50)	0%	0.63
	BRIP1	3	Hu, 2020; Paixão, 2022; Felix, 2022	1530	15203	1.17 (0.15; 9.42)	46%	0.16
	CHEK2	5	Hu, 2020; Molina−Zayas, 2022; Paixão, 2022; Jin, 2023; Evans, 2023	1746	16687	0.68 (0.35; 1.32)	0%	0.70
	PALB2	7	Hu, 2020; Molina−Zayas, 2022; Paixão, 2022; Jin, 2023; Felix, 2022; Evans, 2023; Yang,2017	1902	17982	0.79 (0.37; 1.67)	0%	0.67
	BRCA1	8	Hu, 2020; Sun, 2017; Molina−Zayas, 2022; Paixão, 2022; Felix, 2022; Xiao, 2021; Evans, 2023; Yang,2017	3183	26172	1.46 (0.76; 2.77)	23%	0.24
	SLX4	2	Paixão, 2022; Felix, 2022	31	227	6.89 (0.69; 68.41)	0%	0.61
	MUTYH	2	Paixão, 2022; Jin, 2023	121	348	3.45 (0.37; 31.88)	0%	0.70

3. HR+HER2+ *versus* TNBC	*NBN*	2	Hu, 2020; Felix, 2022	3352	5876	1.08 (0.44; 2.66)	0%	0.58
	BRIP1	2	Hu, 2020; Evans, 2023	3397	3397	0.56 (0.26; 1.21)	0%	0.56
	BRCA2	9	Hu, 2020; Sun, 2017; Molina−Zayas, 2022; Paixão, 2022; Jin, 2023; Felix, 2022; Xiao, 2021; Evans, 2023; Yang,2017	5955	9734	0.78 (0.44; 1.39)	38%	0.12
4. HR+HER2+ *versus* HR+HER2-	*ATM*	5	Hu, 2020; Molina−Zayas, 2022; Paixão, 2022; Felix, 2022; Evans, 2023	3718	16584	2.42 (0.85; 6.87)	39%	0.16
	FANCM	2	Paixão, 2022; Felix, 2022	70	227	1.00 (0.10; 9.75)	0%	0.83
	NBN	2	Hu, 2020; Felix, 2022	3352	15001	1.36 (0.21; 8.99)	42%	0.19
	RAD51D	2	Hu, 2020; Evans, 2023	3266	14752	0.70 (0.12; 4.03)	0%	0.71
	BRIP1	3	Hu, 2020; Paixão, 2022; Felix, 2022	3397	15203	0.85 (0.42; 1.75)	0%	0.97
	CHEK2	5	Hu, 2020; Molina−Zayas, 2022; Paixão, 2022; Jin, 2023; Evans, 2023	3725	16687	1.61 (0.83; 3.12)	0%	0.51
	BRCA2	9	Hu, 2020; Sun, 2017; Molina−Zayas, 2022; Paixão, 2022; Jin, 2023; Felix, 2022; Xiao, 2021; Evans, 2023; Yang,2017	5955	26172	0.63 (0.39; 1.00)	48%	0.05
	PALB2	7	Hu, 2020; Molina−Zayas, 2022; Paixão, 2022; Jin, 2023; Felix, 2022; Evans, 2023; Yang,2017	4050	17982	0.93 (0.53; 1.64)	0%	0.72
	BRCA1	8	Hu, 2020; Sun, 2017; Molina−Zayas, 2022; Paixão, 2022; Felix, 2022; Xiao, 2021; Evans, 2023; Yang, 2017	5955	26172	0.73 (0.53; 1.01)	0%	0.99
5. HR+HER2+ *versus* HR-HER2+	*ATM*	5	Hu, 2020; Molina−Zayas, 2022; Paixão, 2022; Felix, 2022; Evans, 2023	3718	1659	1.59 (0.95; 2.66)	0%	0.96
	NBN	2	Hu, 2020; Felix, 2022	3352	3352	1.63 (0.40; 6.73)	0%	0.92
	MSH6	2	Hu, 202; Molina−Zayas, 2022	1984	878	0.62 (0.02; 17.06)	67%	0.08
	BRIP1	2	Hu, 2020; Felix, 2022	3397	1530	1.10 (0.06; 18.61)	55%	0.14
	BRCA2	7	Hu, 2020; Sun, 2017; Molina−Zayas, 2022; Paixão, 2022; Felix, 2022; Xiao, 2021; Evans, 2023	5955	3183	1.31 (0.93; 1.93)	0%	0.99

	PALB2	5	Hu, 2020; Molina−Zayas, 2022; Paixão, 2022; Jin, 2023; Yang, 2017	4050	1902	1.31 (0.64; 2.66)	0%	0.89
	TP53	3	Hu, 2020; Paixão, 2022; Evans, 2023	4757	2137	0.41 (0.13; 1.31)	9%	0.34
	BRCA1		Hu, 2020; Sun, 2017; Paixão, 2022; Felix, 2022; Xiao, 2021; Evans, 2023; Yang, 2017	5955	3183	0.49 (0.22; 1.09)	17%	0.30
	MUTYH	2	Paixão, 2022; Jin, 2023	82	121	1.23 (0.13; 11.50)	0%	0.75
6. HR+HER2- *versus* TNBC	*FANCM*	3	Paixão, 2022; Felix, 2022; Zhang, 2022	418	170	1.20 (0.19; 7.70)	0%	0.98
	NBN	2	Hu, 2020; Zhang, 2022	15192	5942	1.27 (0.67; 2.39)	0%	0.90
	RAD51D	2	Hu, 2020; Evans, 2023	14943	5842	0.57 (0.04; 7.77)	63%	0.10
	MSH6	2	Hu, 2020; Nikitin, 2020	9642	3611	0.99 (0.50; 1.96)	0%	0.78
	PMS2	3	Paixão, 2022; Zhang, 2022; Nikitin, 2020	518	420	2.52 (0.65; 9.75)	0%	0.89
	BRIP1	5	Hu, 2020; Paixão, 2022; Felix, 2022; Zhang, 2022; Evans, 2023	15394	6031	0.65 (0.41; 1.04)	0%	0.97
	BRCA2	10	Hu, 2020; Sun, 2017; Molina−Zayas, 2022; Paixão, 2022; Jin, 2023; Felix, 2022; Zhang, 2022; Xiao, 2021; Evans, 2023; Yang,2017	26363	9800	1.28 (0.88; 1.86)	56%	0.02
	TP53	6	Hu, 2020; Paixão, 2022; Felix, 2022; Zhang, 2022; Evans, 2023; Yang,2017	21168	8360	1.10 (0.57; 2.11)	0%	0.87

2.1 Supplementary Figures

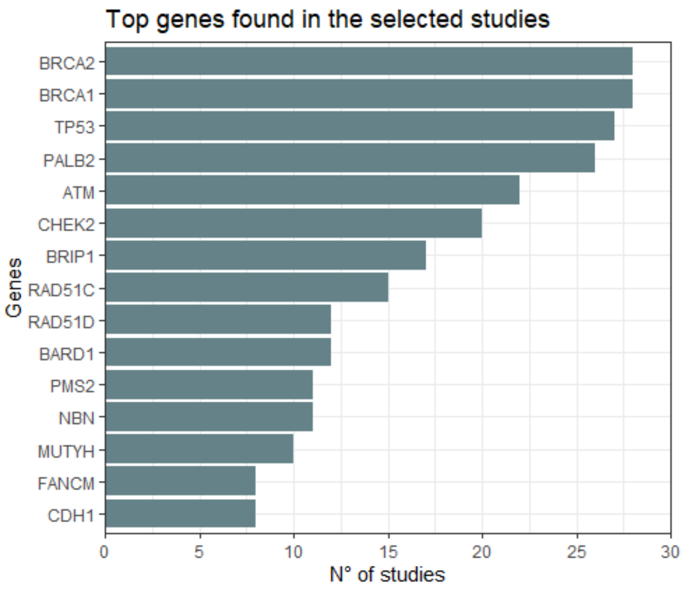

Supplementary Figure 1: Top 15 genes reported in the extracted studies. Representation of the main genes reported among the 32 studies included in the data extraction.

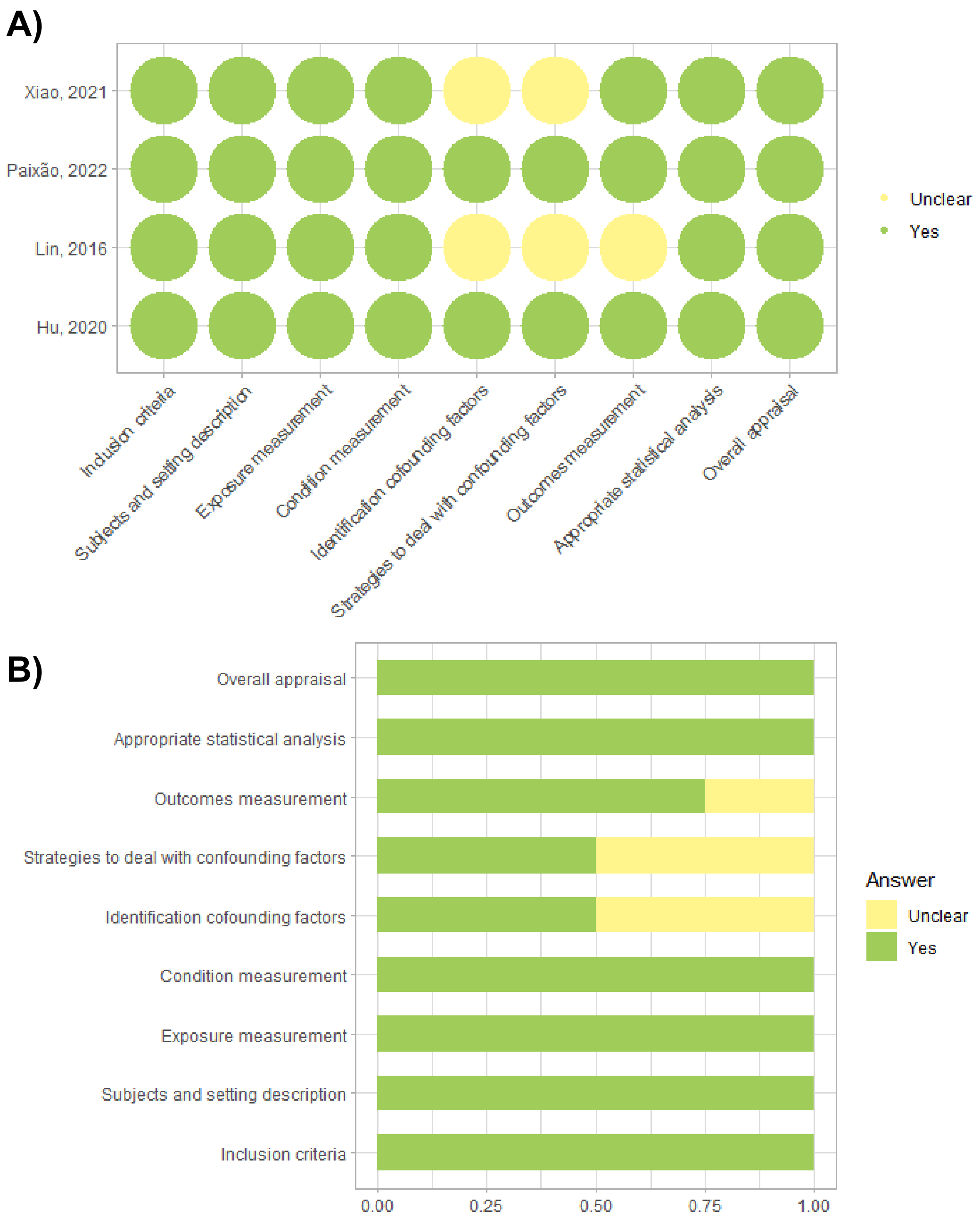

Supplementary Figure 2: Risk of bias assessed for cross-sectional studies using the Joanna Briggs Institute (JBI) Critical Appraisal Tools. A) assessment by study. B) general assessment by evaluated criteria.

Supplementary Material

A)

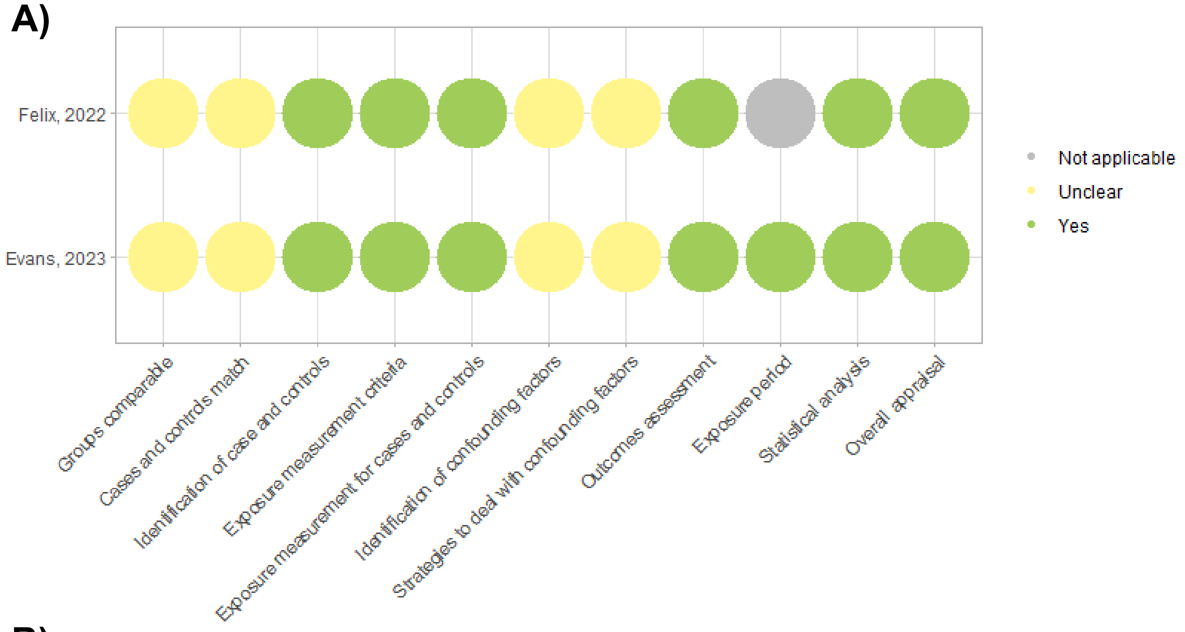

B)

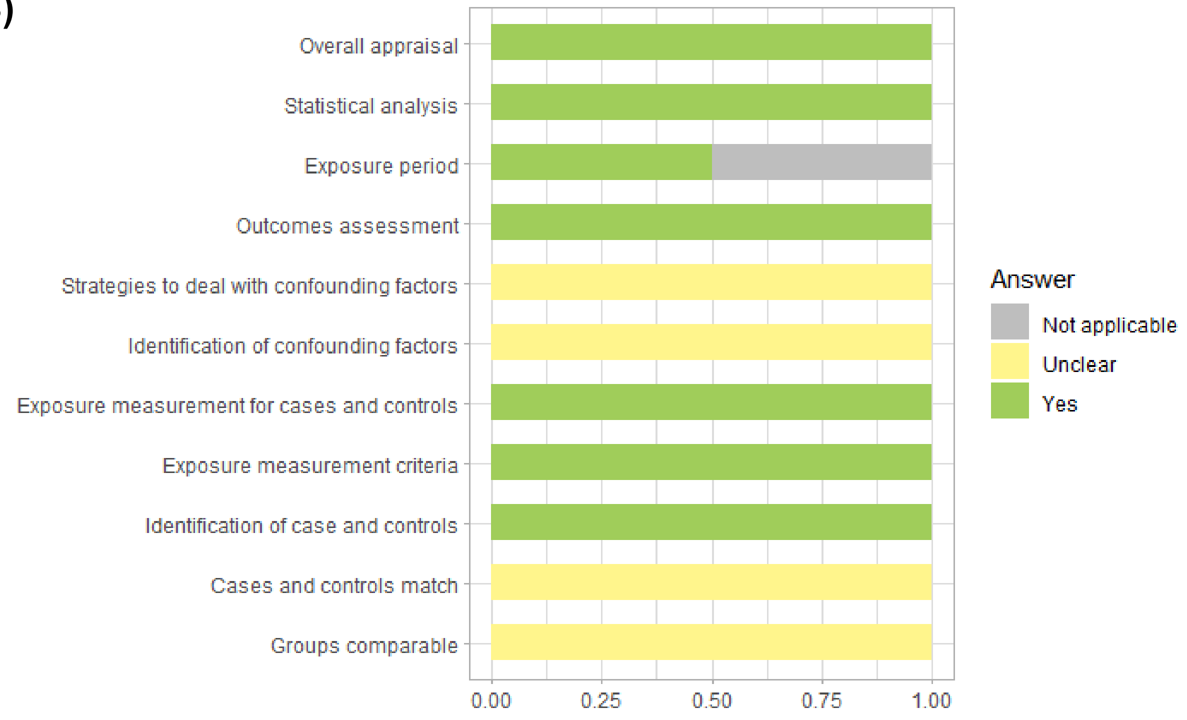

Supplementary Figure 3: Risk of bias assessed for case-control studies using the Joanna Briggs Institute (JBI) Critical Appraisal Tools. A: assessment by study. B: general assessment by evaluated criteria.

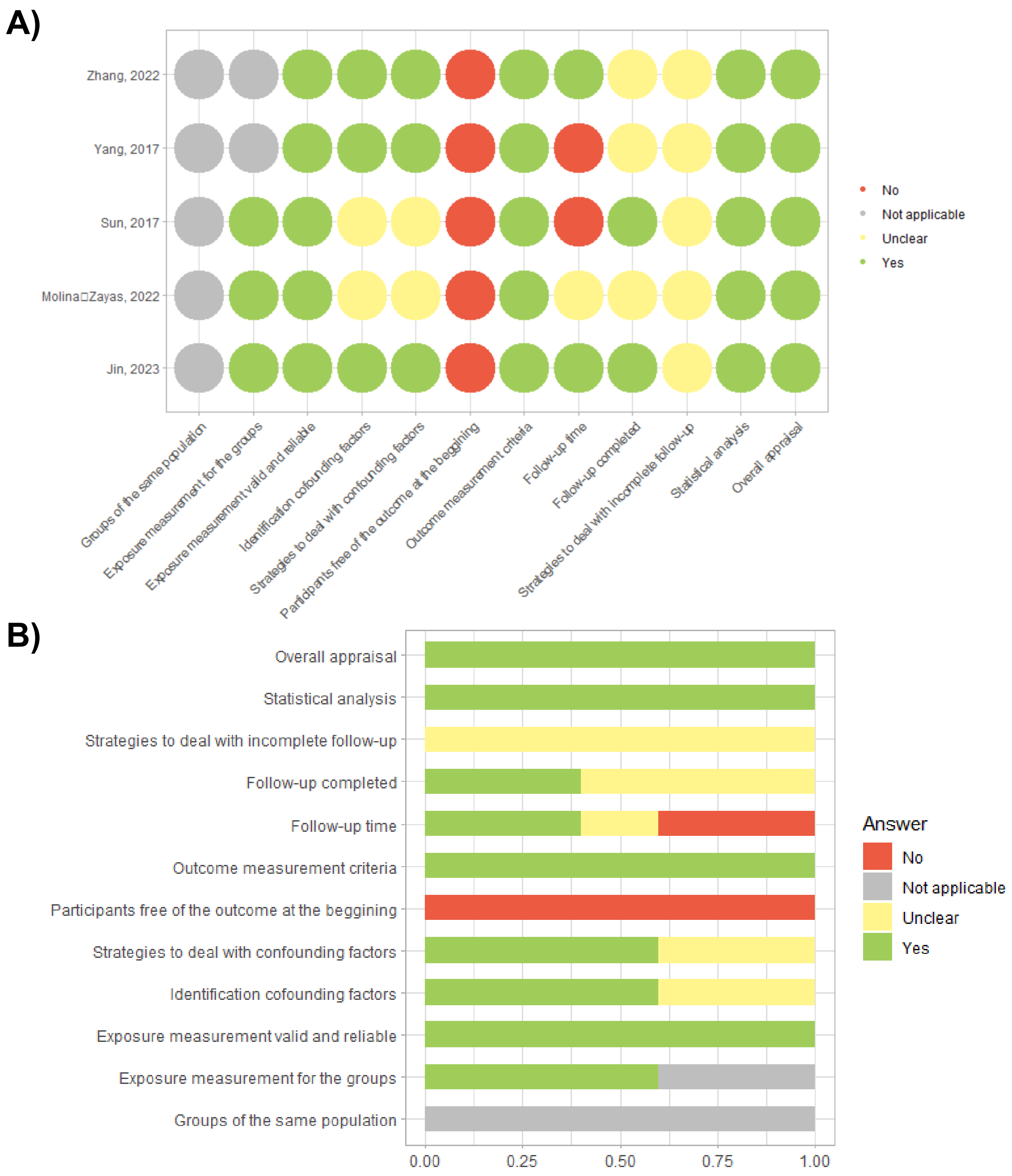

Supplementary Figure 4: Risk of bias assessed for cohort studies using the Joanna Briggs Institute (JBI) Critical Appraisal Tools. A: assessment by study. B: general assessment by evaluated criteria.

HR-HER2+ x TNBC

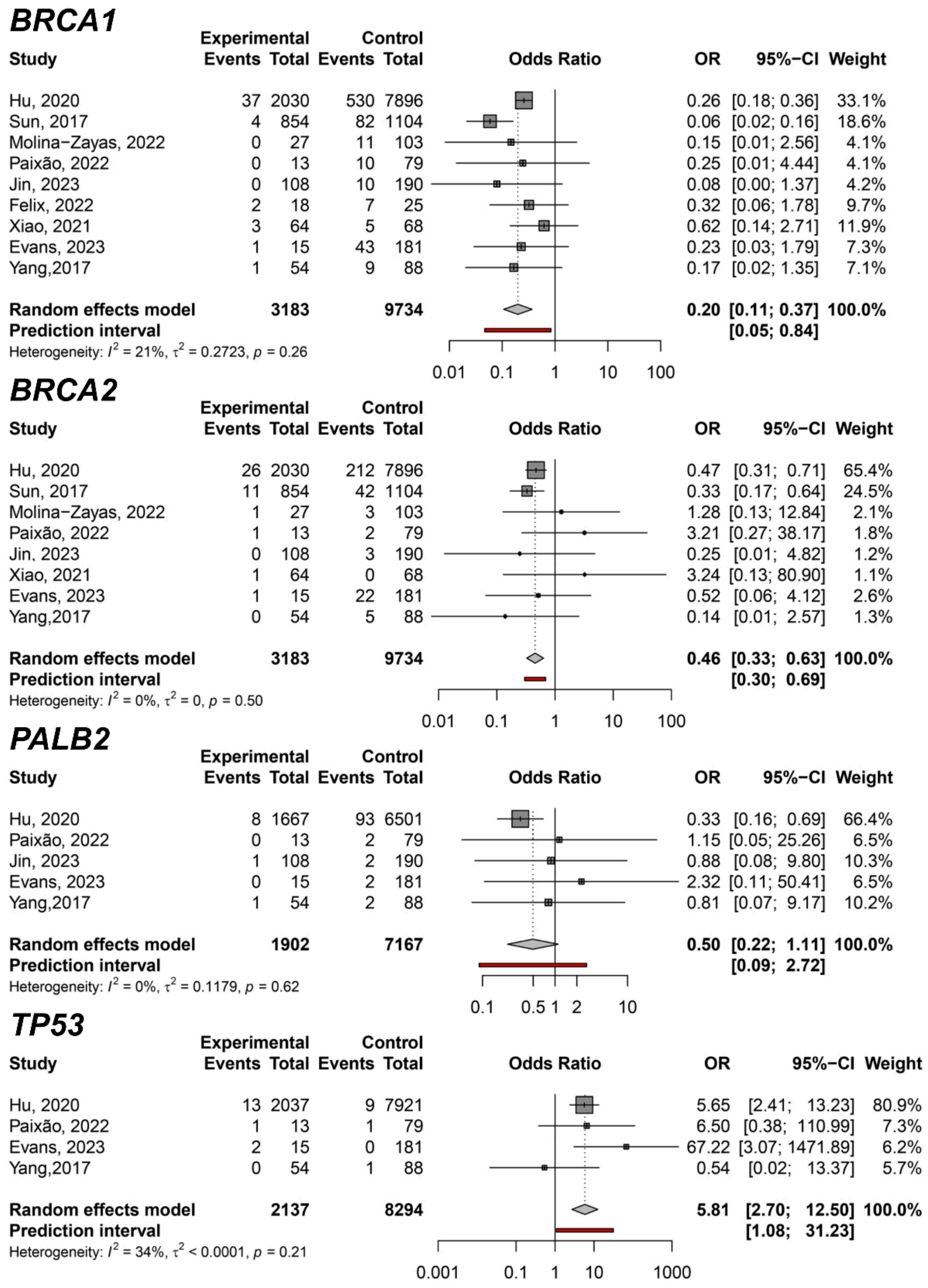

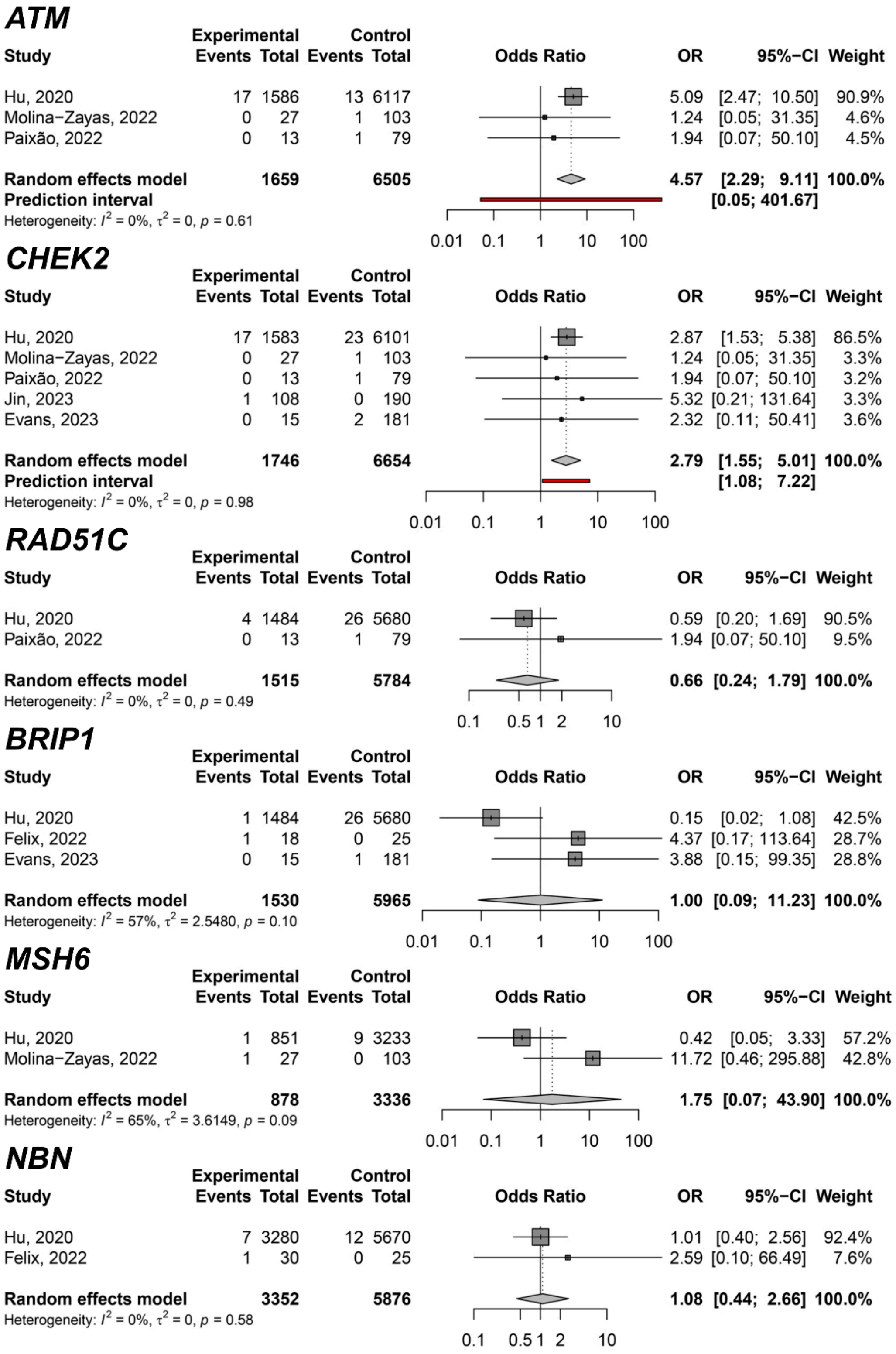

Supplementary figure 5. Forest plots showing the predisposition to HR-HER2+ (cases) breast cancer subtype compared to TNBC (controls).

Supplementary Material

HR-HER2+ x HR+HER2-

BRCA1

Study	Experimental Events	Experimental Total	Control Events	Control Total	OR	95%−CI	Weight
Hu, 2020	37	2030	185	20275	2.02	[1.41; 2.88]	35.7%
Sun, 2017	4	854	47	4438	0.44	[0.16; 1.22]	19.9%
Molina−Zayas, 2022	0	27	4	324	1.29	[0.07; 24.68]	4.3%
Paixão, 2022	0	13	4	155	1.25	[0.06; 24.41]	4.2%
Felix, 2022	2	18	2	72	4.38	[0.57; 33.44]	8.0%
Xiao, 2021	3	64	6	321	2.58	[0.63; 10.60]	13.7%
Evans, 2023	1	15	11	151	0.91	[0.11; 7.57]	7.5%
Yang,2017	1	54	3	243	1.51	[0.15; 14.80]	6.6%
**Random effects model**		**3183**		**26172**	**1.46**	**[0.76; 2.77]**	**100.0%**
**Prediction interval**						**[0.32; 6.55]**	

Heterogeneity: $I^2 = 23\%$, $\tau^2 = 0.2694$, $p = 0.24$

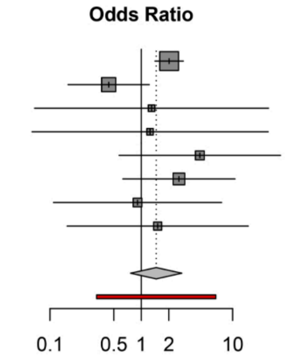

BRCA2

Study	Experimental Events	Experimental Total	Control Events	Control Total	OR	95%−CI	Weight
Hu, 2020	26	2030	424	20275	0.61	[0.41; 0.90]	42.1%
Sun, 2017	11	854	190	4438	0.29	[0.16; 0.54]	30.3%
Molina−Zayas, 2022	1	27	22	324	0.53	[0.07; 4.08]	5.1%
Paixão, 2022	1	13	5	155	2.50	[0.27; 23.16]	4.4%
Jin, 2023	0	108	8	193	0.10	[0.01; 1.76]	2.7%
Felix, 2022	0	18	6	72	0.28	[0.01; 5.14]	2.6%
Xiao, 2021	1	64	15	321	0.32	[0.04; 2.50]	5.1%
Evans, 2023	1	15	32	151	0.27	[0.03; 2.10]	5.0%
Yang,2017	0	54	10	243	0.20	[0.01; 3.54]	2.7%
**Random effects model**		**3183**		**26172**	**0.43**	**[0.27; 0.70]**	**100.0%**
**Prediction interval**						**[0.17; 1.12]**	

Heterogeneity: $I^2 = 1\%$, $\tau^2 = 0.1025$, $p = 0.43$

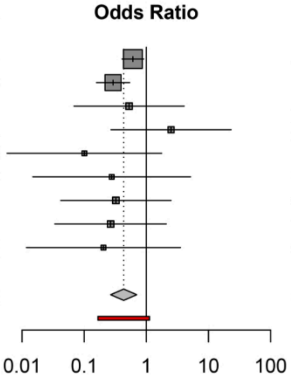

PALB2

Study	Experimental Events	Experimental Total	Control Events	Control Total	OR	95%−CI	Weight
Hu, 2020	8	1667	155	16844	0.52	[0.25; 1.06]	60.4%
Molina−Zayas, 2022	0	27	4	324	1.29	[0.07; 24.68]	6.2%
Paixão, 2022	0	13	1	155	3.81	[0.15; 98.25]	5.1%
Jin, 2023	1	108	3	193	0.59	[0.06; 5.76]	10.1%
Felix, 2022	0	18	2	72	0.76	[0.04; 16.57]	5.7%
Evans, 2023	0	15	2	151	1.93	[0.09; 42.02]	5.7%
Yang,2017	1	54	1	243	4.57	[0.28; 74.17]	6.9%
**Random effects model**		**1902**		**17982**	**0.79**	**[0.37; 1.67]**	**100.0%**
**Prediction interval**						**[0.21; 2.91]**	

Heterogeneity: $I^2 = 0\%$, $\tau^2 = 0.1110$, $p = 0.67$

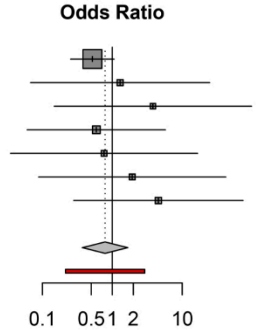

TP53

Study	Experimental Events	Experimental Total	Control Events	Control Total	OR	95%−CI	Weight
Hu, 2020	13	2037	25	20356	5.22	[2.67; 10.23]	78.8%
Paixão, 2022	1	13	2	155	6.37	[0.54; 75.47]	5.8%
Felix, 2022	0	18	1	72	1.29	[0.05; 32.93]	3.4%
Evans, 2023	2	15	2	151	11.46	[1.49; 88.17]	8.5%
Yang,2017	0	54	1	243	1.48	[0.06; 36.90]	3.4%
**Random effects model**		**2137**		**20977**	**5.16**	**[2.84; 9.37]**	**100.0%**
**Prediction interval**						**[1.96; 13.59]**	

Heterogeneity: $I^2 = 0\%$, $\tau^2 = 0$, $p = 0.75$

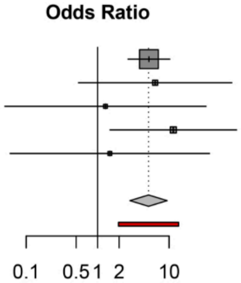

ATM

Study	Experimental Events	Experimental Total	Control Events	Control Total	OR	95%-CI	Weight
Hu, 2020	17	1586	179	15882	0.95	[0.58; 1.57]	90.9%
Molina−Zayas, 2022	0	27	4	324	1.29	[0.07; 24.68]	2.6%
Paixão, 2022	0	13	1	155	3.81	[0.15; 98.25]	2.2%
Felix, 2022	0	18	1	72	1.29	[0.05; 32.93]	2.2%
Evans, 2023	0	15	1	151	3.24	[0.13; 82.89]	2.2%
**Random effects model**		**1659**		**16584**	**1.02**	**[0.63; 1.64]**	**100.0%**
**Prediction interval**						**[0.47; 2.21]**	

Heterogeneity: $I^2 = 0\%$, $\tau^2 = 0$, $p = 0.87$

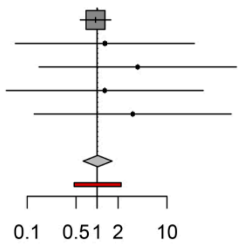

CHEK2

Study	Experimental Events	Experimental Total	Control Events	Control Total	OR	95%-CI	Weight
Hu, 2020	17	1583	305	15864	0.55	[0.34; 0.91]	80.9%
Molina−Zayas, 2022	0	27	2	324	2.35	[0.11; 50.08]	4.5%
Paixão, 2022	0	13	2	155	2.27	[0.10; 49.82]	4.4%
Jin, 2023	1	108	1	193	1.79	[0.11; 28.98]	5.4%
Evans, 2023	0	15	5	151	0.86	[0.05; 16.29]	4.8%
**Random effects model**		**1746**		**16687**	**0.68**	**[0.35; 1.32]**	**100.0%**
**Prediction interval**						**[0.17; 2.73]**	

Heterogeneity: $I^2 = 0\%$, $\tau^2 = 0.0767$, $p = 0.70$

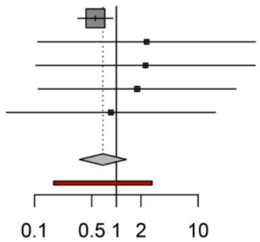

BRIP1

Study	Experimental Events	Experimental Total	Control Events	Control Total	OR	95%-CI	Weight
Hu, 2020	1	1484	43	14825	0.23	[0.03; 1.68]	43.1%
Paixão, 2022	0	13	1	155	3.81	[0.15; 98.25]	26.0%
Felix, 2022	1	18	1	72	4.18	[0.25; 70.20]	30.8%
**Random effects model**		**1530**		**15203**	**1.17**	**[0.15; 9.42]**	**100.0%**
**Prediction interval**						**[0.00; 1533578550.38]**	

Heterogeneity: $I^2 = 46\%$, $\tau^2 = 1.5982$, $p = 0.16$

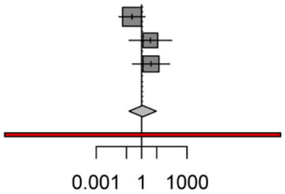

MSH6

Study	Experimental Events	Experimental Total	Control Events	Control Total	OR	95%-CI	Weight
Hu, 2020	1	851	24	9146	0.45	[0.06; 3.31]	54.3%
Molina−Zayas, 2022	1	27	0	324	36.74	[1.46; 924.05]	45.7%
**Random effects model**		**878**		**9470**	**3.36**	**[0.05; 248.43]**	**100.0%**

Heterogeneity: $I^2 = 81\%$, $\tau^2 = 7.8427$, $p = 0.02$

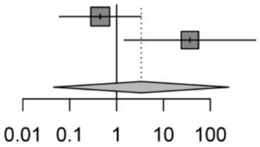

RAD51D

Study	Experimental Events	Experimental Total	Control Events	Control Total	OR	95%-CI	Weight
Hu, 2020	1	1460	8	14601	1.25	[0.16; 10.00]	70.9%
Evans, 2023	0	15	1	151	3.24	[0.13; 82.89]	29.1%
**Random effects model**		**1475**		**14752**	**1.65**	**[0.29; 9.50]**	**100.0%**

Heterogeneity: $I^2 = 0\%$, $\tau^2 = 0$, $p = 0.63$

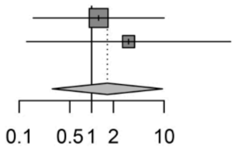

MUTYH

Study	Experimental Events	Experimental Total	Control Events	Control Total	OR	95%-CI	Weight
Paixão, 2022	0	13	2	155	2.27	[0.10; 49.82]	51.9%
Jin, 2023	1	108	0	193	5.40	[0.22; 133.71]	48.1%
**Random effects model**		**121**		**348**	**3.45**	**[0.37; 31.88]**	**100.0%**

Heterogeneity: $I^2 = 0\%$, $\tau^2 = 0$, $p = 0.70$

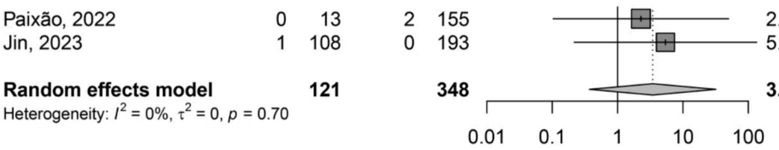

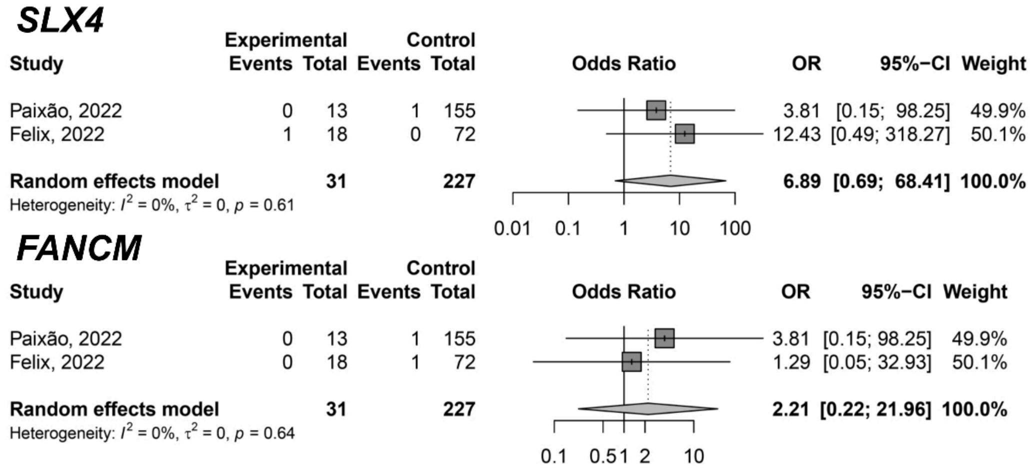

Supplementary figure 6. Forest plots showing the predisposition to HR-HER2+ (cases) breast cancer subtype compared to HR+HER2- (controls).

HR+HER2+ x TNBC

BRCA1

Study	Experimental Events	Experimental Total	Control Events	Control Total	OR	95%−CI	Weight
Hu, 2020	32	4562	530	7896	0.10	[0.07; 0.14]	71.9%
Sun, 2017	8	965	82	1104	0.10	[0.05; 0.22]	17.3%
Molina−Zayas, 2022	0	99	11	103	0.04	[0.00; 0.70]	1.1%
Paixão, 2022	1	40	10	79	0.18	[0.02; 1.43]	2.1%
Jin, 2023	0	42	10	190	0.20	[0.01; 3.52]	1.1%
Felix, 2022	0	30	7	25	0.04	[0.00; 0.75]	1.1%
Xiao, 2021	1	111	5	68	0.11	[0.01; 1.00]	2.0%
Evans, 2023	1	42	43	181	0.08	[0.01; 0.59]	2.3%
Yang,2017	0	64	9	88	0.06	[0.00; 1.14]	1.1%
**Random effects model**		**5955**		**9734**	**0.10**	**[0.07; 0.13]**	**100.0%**
**Prediction interval**						**[0.07; 0.14]**	

Heterogeneity: $I^2 = 0\%$, $\tau^2 = 0$, $p = 0.99$

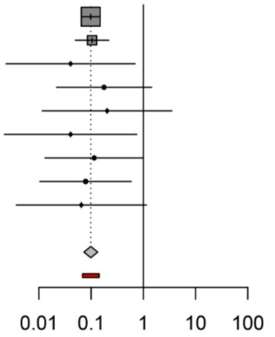

BRCA2

Study	Experimental Events	Experimental Total	Control Events	Control Total	OR	95%−CI	Weight
Hu, 2020	76	4562	212	7896	0.61	[0.47; 0.80]	31.4%
Sun, 2017	18	965	42	1104	0.48	[0.27; 0.84]	25.5%
Molina−Zayas, 2022	6	99	3	103	2.15	[0.52; 8.85]	11.1%
Paixão, 2022	4	40	2	79	4.28	[0.75; 24.44]	8.3%
Jin, 2023	0	42	3	190	0.63	[0.03; 12.43]	3.4%
Felix, 2022	2	30	0	25	4.47	[0.20; 97.63]	3.2%
Xiao, 2021	2	111	0	68	3.13	[0.15; 66.13]	3.2%
Evans, 2023	2	42	22	181	0.36	[0.08; 1.60]	10.4%
Yang,2017	0	64	5	88	0.12	[0.01; 2.17]	3.5%
**Random effects model**		**5955**		**9734**	**0.78**	**[0.44; 1.39]**	**100.0%**
**Prediction interval**						**[0.19; 3.14]**	

Heterogeneity: $I^2 = 38\%$, $\tau^2 = 0.2584$, $p = 0.12$

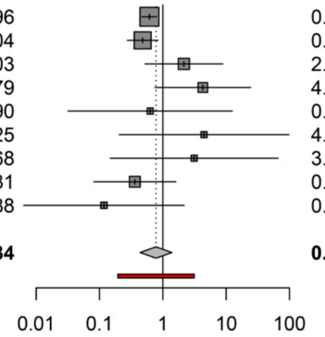

PALB2

Study	Experimental Events	Experimental Total	Control Events	Control Total	OR	95%−CI	Weight
Hu, 2020	26	3733	93	6501	0.48	[0.31; 0.75]	86.5%
Molina−Zayas, 2022	3	99	0	103	7.51	[0.38; 147.24]	2.5%
Paixão, 2022	1	40	2	79	0.99	[0.09; 11.23]	3.8%
Jin, 2023	0	42	2	190	0.89	[0.04; 18.82]	2.4%
Evans, 2023	0	42	2	181	0.84	[0.04; 17.92]	2.4%
Yang,2017	0	64	2	88	0.27	[0.01; 5.68]	2.4%
**Random effects model**		**4050**		**7167**	**0.54**	**[0.34; 0.87]**	**100.0%**
**Prediction interval**						**[0.25; 1.16]**	

Heterogeneity: $I^2 = 0\%$, $\tau^2 = 0.0180$, $p = 0.57$

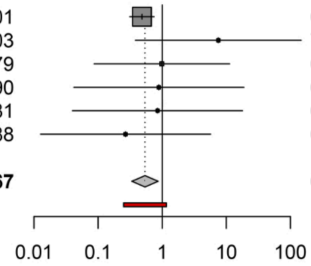

TP53

Study	Experimental Events	Experimental Total	Control Events	Control Total	OR	95%−CI	Weight
Hu, 2020	19	4581	9	7921	3.66	[1.66; 8.10]	84.6%
Paixão, 2022	0	40	1	79	0.65	[0.03; 16.22]	5.1%
Evans, 2023	1	42	0	181	13.12	[0.53; 327.83]	5.1%
Yang,2017	0	64	1	88	0.45	[0.02; 11.28]	5.2%
**Random effects model**		**4757**		**8294**	**3.21**	**[1.55; 6.67]**	**100.0%**
**Prediction interval**						**[0.65; 15.95]**	

Heterogeneity: $I^2 = 7\%$, $\tau^2 < 0.0001$, $p = 0.36$

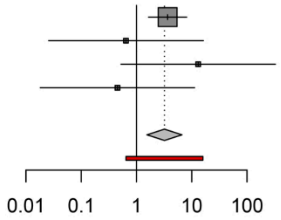

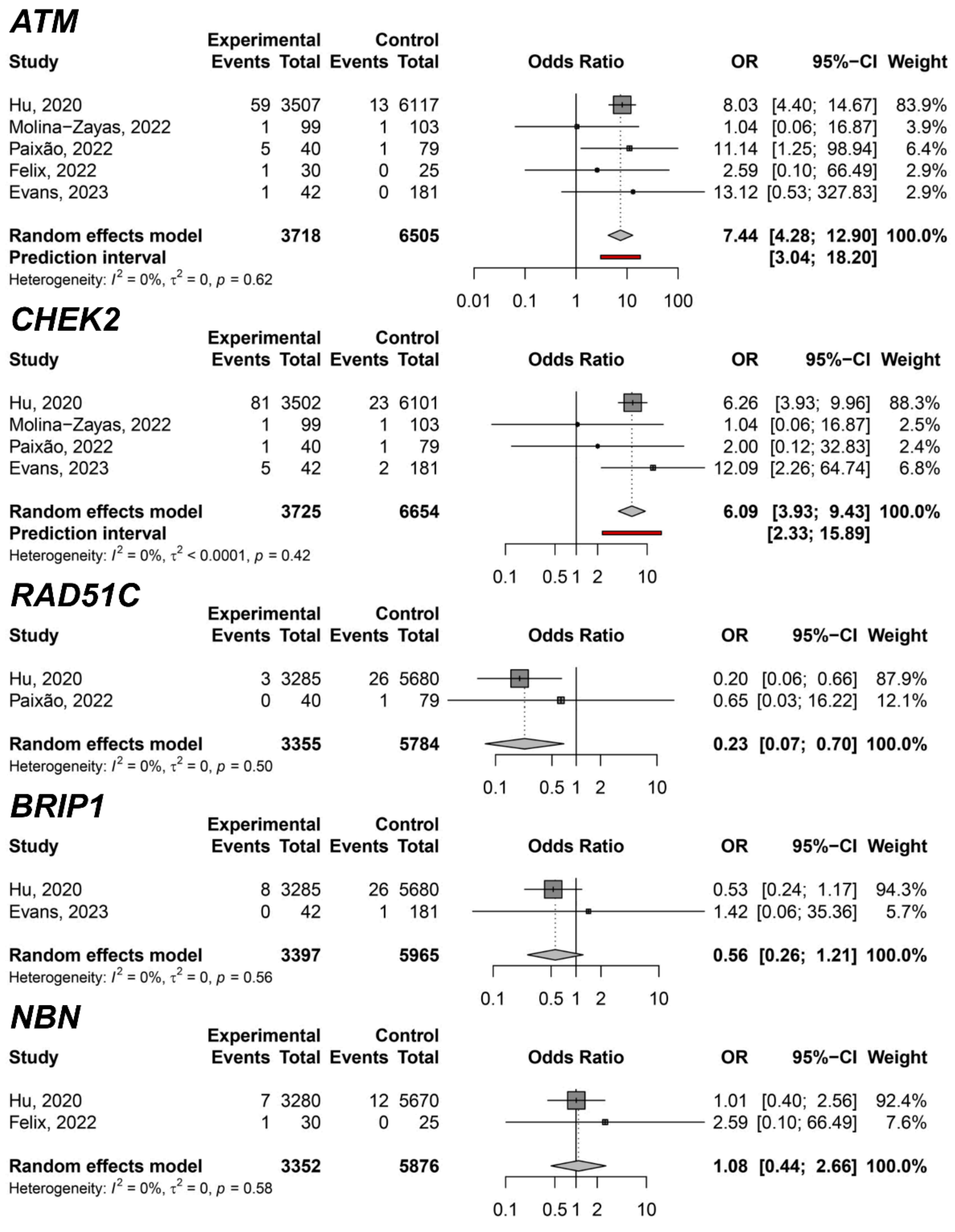

Supplementary figure 7. Forest plots showing the predisposition to HR+HER2+ (cases) breast cancer subtype compared to TNBC (controls).

HR+HER2+ x HR+HER2-

BRCA1

Study	Experimental Events	Experimental Total	Control Events	Control Total	OR	95%-CI	Weight
Hu, 2020	32	4562	185	20275	0.77	[0.53; 1.12]	71.9%
Sun, 2017	8	965	47	4438	0.78	[0.37; 1.66]	18.0%
Molina−Zayas, 2022	0	99	4	324	0.36	[0.02; 6.71]	1.2%
Paixão, 2022	1	40	4	155	0.97	[0.11; 8.91]	2.1%
Felix, 2022	0	30	2	72	0.46	[0.02; 9.92]	1.1%
Xiao, 2021	1	111	6	321	0.48	[0.06; 4.01]	2.3%
Evans, 2023	1	42	11	151	0.31	[0.04; 2.48]	2.4%
Yang,2017	0	64	3	243	0.53	[0.03; 10.44]	1.2%
**Random effects model**		**5955**		**26172**	**0.73**	**[0.53; 1.01]**	**100.0%**
**Prediction interval**						**[0.49; 1.10]**	

Heterogeneity: I^2 = 0%, τ^2 = 0, p = 0.99

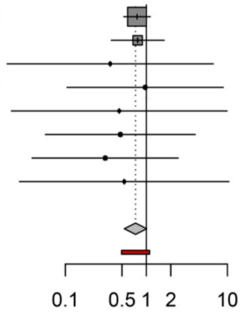

BRCA2

Study	Experimental Events	Experimental Total	Control Events	Control Total	OR	95%-CI	Weight
Hu, 2020	76	4562	424	20275	0.79	[0.62; 1.01]	28.5%
Sun, 2017	18	965	190	4438	0.42	[0.26; 0.69]	23.1%
Molina−Zayas, 2022	6	99	22	324	0.89	[0.35; 2.25]	13.8%
Paixão, 2022	4	40	5	155	3.33	[0.85; 13.04]	8.5%
Jin, 2023	0	42	8	193	0.26	[0.01; 4.54]	2.4%
Felix, 2022	2	30	6	72	0.79	[0.15; 4.13]	6.3%
Xiao, 2021	2	111	15	321	0.37	[0.08; 1.66]	7.4%
Evans, 2023	2	42	32	151	0.19	[0.04; 0.81]	7.6%
Yang,2017	0	64	10	243	0.17	[0.01; 2.98]	2.5%
**Random effects model**		**5955**		**26172**	**0.63**	**[0.39; 1.00]**	**100.0%**
**Prediction interval**						**[0.20; 1.99]**	

Heterogeneity: I^2 = 48%, τ^2 = 0.1824, p = 0.05

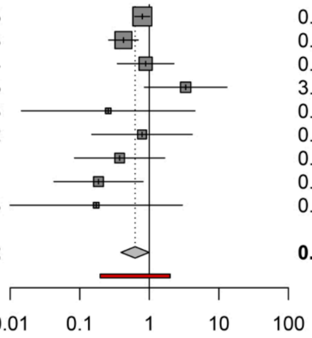

PALB2

Study	Experimental Events	Experimental Total	Control Events	Control Total	OR	95%-CI	Weight
Hu, 2020	26	3733	155	16844	0.76	[0.50; 1.15]	70.3%
Molina−Zayas, 2022	3	99	4	324	2.50	[0.55; 11.36]	12.5%
Paixão, 2022	1	40	1	155	3.95	[0.24; 64.55]	4.0%
Jin, 2023	0	42	3	193	0.64	[0.03; 12.63]	3.5%
Felix, 2022	0	30	2	72	0.46	[0.02; 9.92]	3.3%
Evans, 2023	0	42	2	151	0.70	[0.03; 14.94]	3.3%
Yang,2017	0	64	1	243	1.25	[0.05; 31.13]	3.0%
**Random effects model**		**4050**		**17982**	**0.93**	**[0.53; 1.64]**	**100.0%**
**Prediction interval**						**[0.33; 2.58]**	

Heterogeneity: I^2 = 0%, τ^2 = 0.0739, p = 0.72

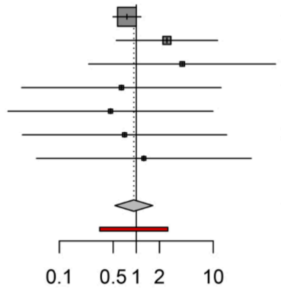

TP53

Study	Experimental Events	Experimental Total	Control Events	Control Total	OR	95%-CI	Weight
Hu, 2020	19	4581	25	20356	3.39	[1.86; 6.16]	82.8%
Paixão, 2022	0	40	2	155	0.76	[0.04; 16.10]	3.9%
Felix, 2022	0	30	1	72	0.78	[0.03; 19.73]	3.5%
Evans, 2023	1	42	2	151	1.82	[0.16; 20.54]	6.2%
Yang,2017	0	64	1	243	1.25	[0.05; 31.13]	3.6%
**Random effects model**		**4757**		**20977**	**2.82**	**[1.53; 5.18]**	**100.0%**
**Prediction interval**						**[0.93; 8.49]**	

Heterogeneity: I^2 = 0%, τ^2 = 0.0238, p = 0.73

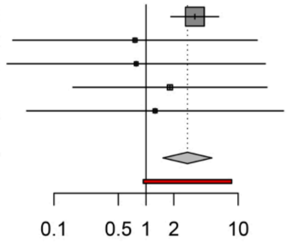

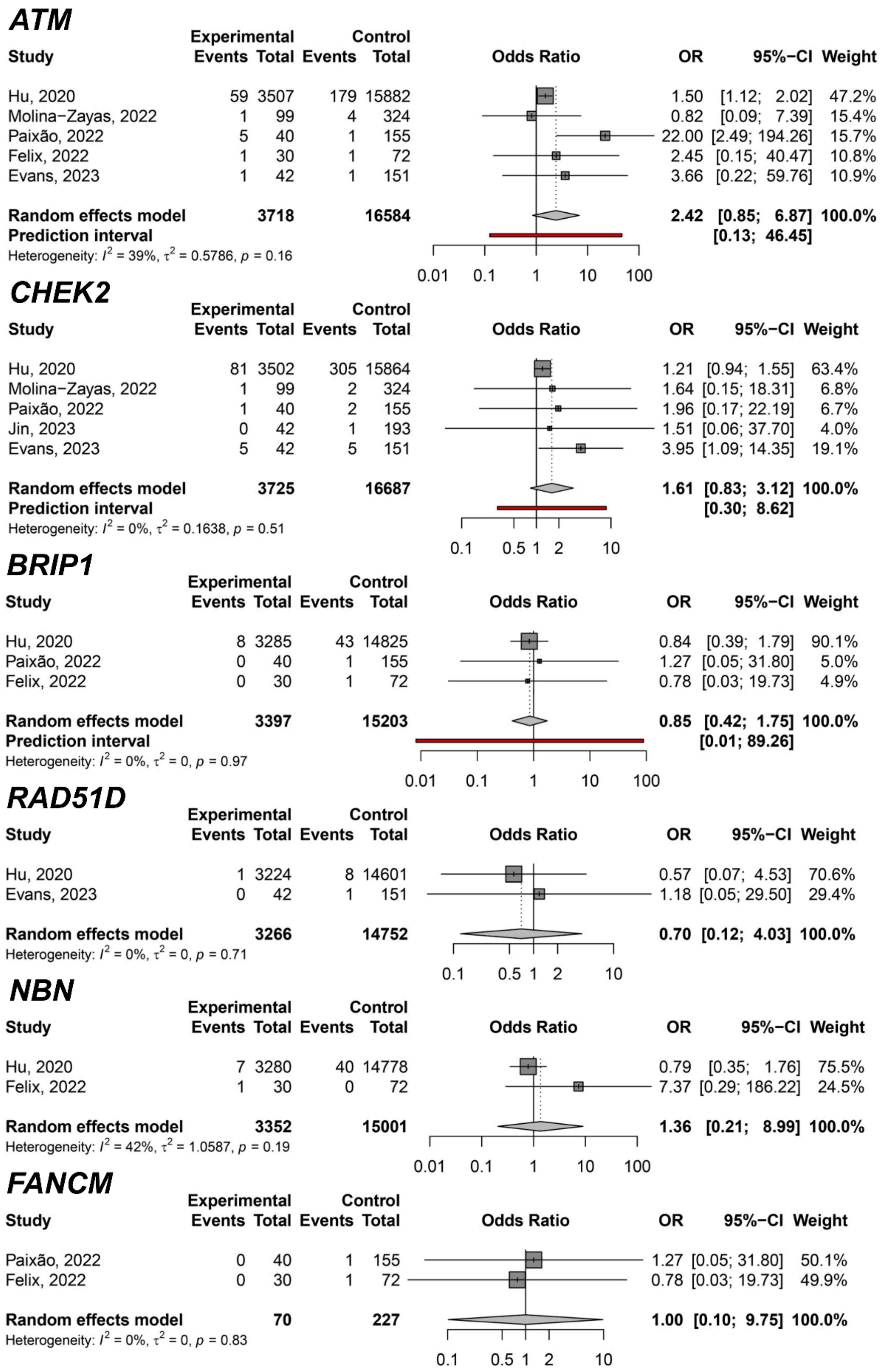

Supplementary figure 8. Forest plots showing the predisposition to HR+HER2+ (cases) breast cancer subtype compared to HR+HER2- (controls).

HR+HER2+ x HR-HER2+

BRCA1

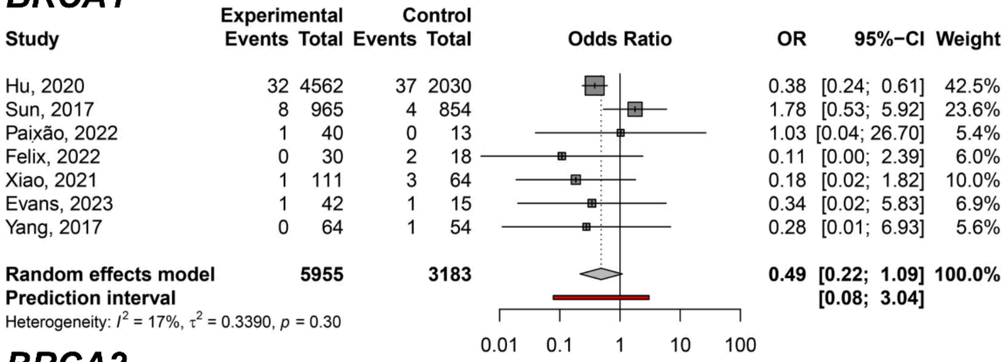

BRCA2

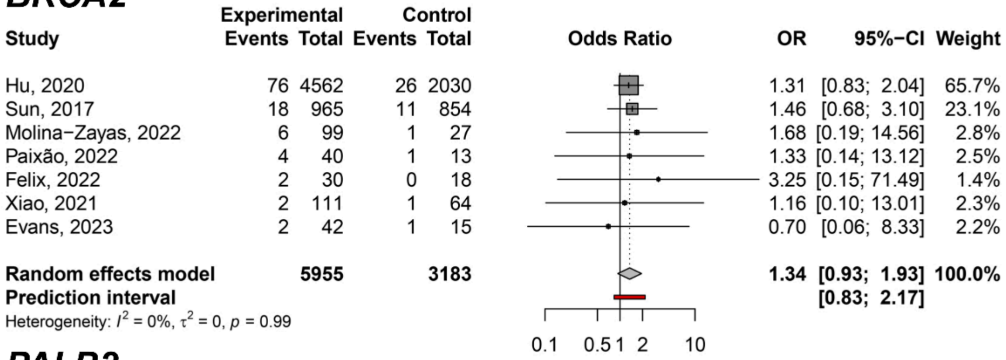

PALB2

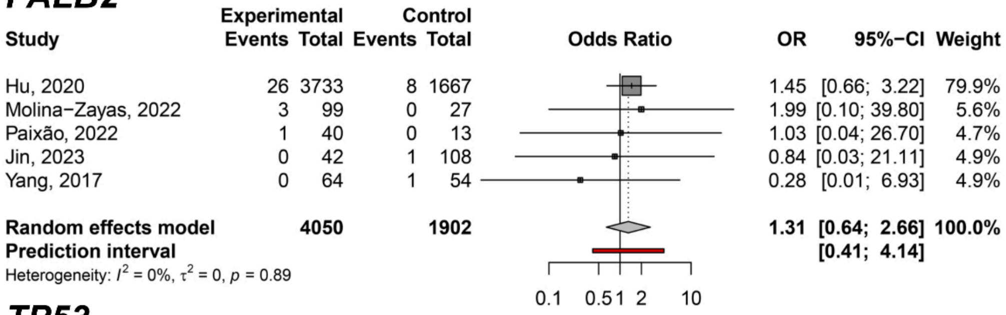

TP53

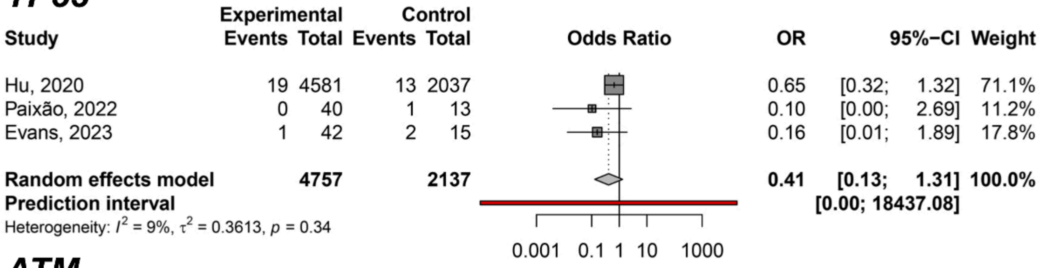

ATM

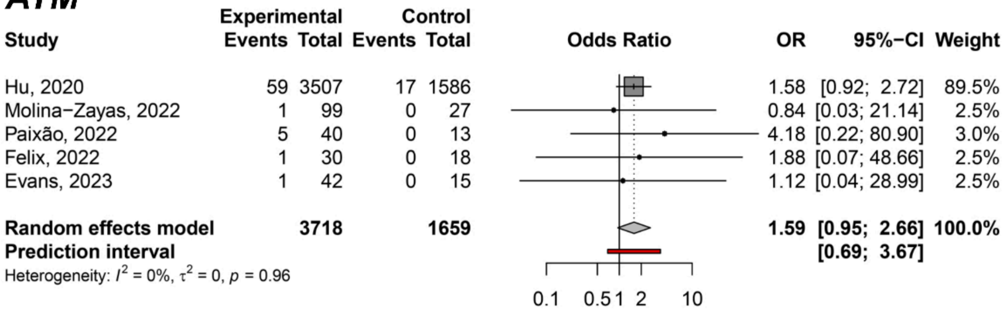

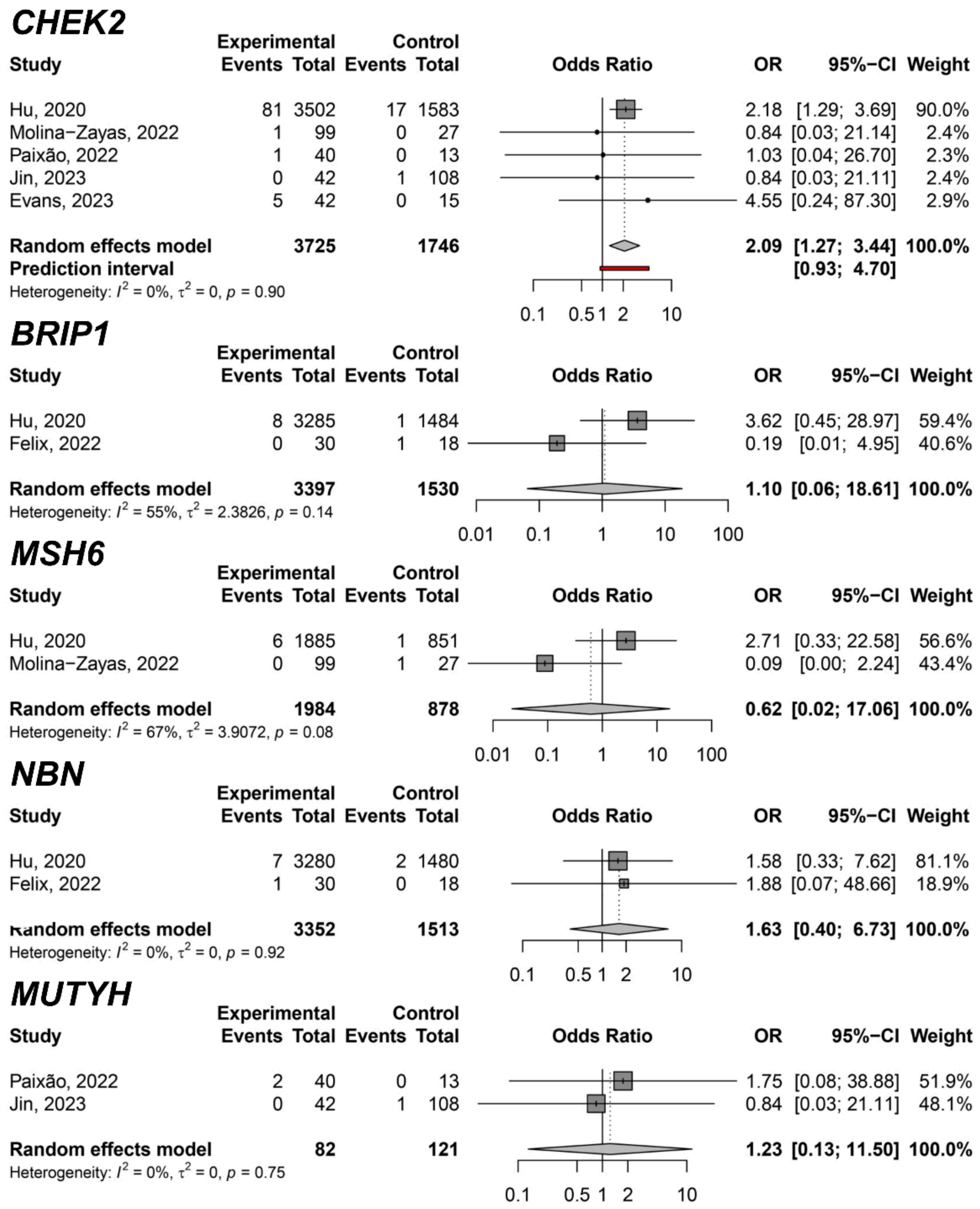

Supplementary figure 9. Forest plots showing the predisposition to HR+HER2+ (cases) breast cancer subtype compared to HR-HER2+ (controls).

HR+HER2- x TNBC

BRCA1

Study	Experimental Events	Experimental Total	Control Events	Control Total	OR	95%-CI	Weight
Hu, 2020	185	20275	530	7896	0.13	[0.11; 0.15]	72.6%
Sun, 2017	47	4438	82	1104	0.13	[0.09; 0.19]	15.6%
Molina−Zayas, 2022	4	324	11	103	0.10	[0.03; 0.34]	1.5%
Paixão, 2022	4	155	10	79	0.18	[0.06; 0.60]	1.5%
Jin, 2023	0	193	10	190	0.04	[0.00; 0.76]	0.3%
Felix, 2022	2	72	7	25	0.07	[0.01; 0.38]	0.8%
Zhang, 2022	3	191	5	66	0.19	[0.05; 0.84]	1.0%
Xiao, 2021	6	321	5	68	0.24	[0.07; 0.81]	1.4%
Evans, 2023	11	151	43	181	0.25	[0.12; 0.51]	4.2%
Yang,2017	3	243	9	88	0.11	[0.03; 0.42]	1.2%
**Random effects model**		**26363**		**9800**	**0.13**	**[0.12; 0.15]**	**100.0%**
**Prediction interval**						**[0.11; 0.16]**	

Heterogeneity: $I^2 = 0\%$, $\tau^2 = 0$, $p = 0.73$

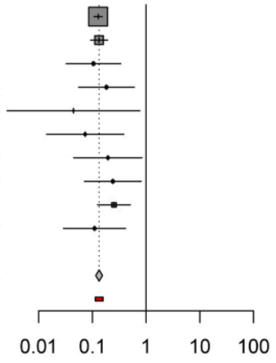

BRCA2

Study	Experimental Events	Experimental Total	Control Events	Control Total	OR	95%-CI	Weight
Hu, 2020	424	20275	212	7896	0.77	[0.66; 0.91]	26.2%
Sun, 2017	190	4438	42	1104	1.13	[0.80; 1.59]	22.5%
Molina−Zayas, 2022	22	324	3	103	2.43	[0.71; 8.29]	7.0%
Paixão, 2022	5	155	2	79	1.28	[0.24; 6.77]	4.3%
Jin, 2023	8	193	3	190	2.70	[0.70; 10.32]	6.1%
Felix, 2022	6	72	0	25	4.98	[0.27; 91.73]	1.6%
Zhang, 2022	11	191	3	66	1.28	[0.35; 4.75]	6.3%
Xiao, 2021	15	321	0	68	6.93	[0.41; 117.20]	1.6%
Evans, 2023	32	151	22	181	1.94	[1.07; 3.51]	16.3%
Yang,2017	10	243	5	88	0.71	[0.24; 2.15]	8.1%
**Random effects model**		**26363**		**9800**	**1.28**	**[0.88; 1.86]**	**100.0%**
**Prediction interval**						**[0.50; 3.29]**	

Heterogeneity: $I^2 = 56\%$, $\tau^2 = 0.1316$, $p = 0.02$

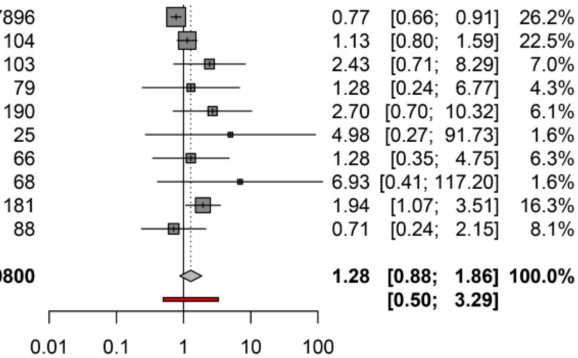

PALB2

Study	Experimental Events	Experimental Total	Control Events	Control Total	OR	95%-CI	Weight
Hu, 2020	155	16844	93	6501	0.64	[0.49; 0.83]	93.0%
Molina−Zayas, 2022	4	324	0	103	2.91	[0.16; 54.44]	0.7%
Paixão, 2022	1	155	2	79	0.25	[0.02; 2.80]	1.1%
Jin, 2023	3	193	2	190	1.48	[0.25; 8.98]	1.9%
Felix, 2022	2	72	0	25	1.81	[0.08; 38.96]	0.7%
Evans, 2023	2	151	2	181	1.20	[0.17; 8.63]	1.6%
Yang,2017	1	243	2	88	0.18	[0.02; 1.98]	1.1%
**Random effects model**		**17982**		**7167**	**0.65**	**[0.51; 0.84]**	**100.0%**
**Prediction interval**						**[0.47; 0.91]**	

Heterogeneity: $I^2 = 0\%$, $\tau^2 = 0$, $p = 0.63$

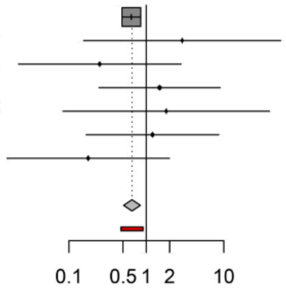

TP53

Study	Experimental Events	Experimental Total	Control Events	Control Total	OR	95%-CI	Weight
Hu, 2020	25	20356	9	7921	1.08	[0.50; 2.32]	74.1%
Paixão, 2022	2	155	1	79	1.02	[0.09; 11.42]	7.4%
Felix, 2022	1	72	0	25	1.07	[0.04; 27.11]	4.1%
Zhang, 2022	1	191	0	66	1.05	[0.04; 26.02]	4.2%
Evans, 2023	2	151	0	181	6.07	[0.29; 127.42]	4.6%
Yang,2017	1	243	1	88	0.36	[0.02; 5.81]	5.6%
**Random effects model**		**21168**		**8360**	**1.10**	**[0.57; 2.11]**	**100.0%**
**Prediction interval**						**[0.43; 2.77]**	

Heterogeneity: $I^2 = 0\%$, $\tau^2 = 0$, $p = 0.87$

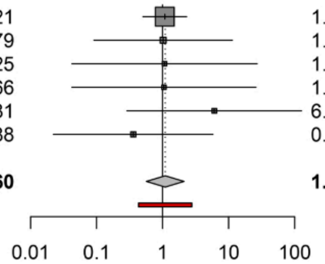

ATM

Study	Experimental Events	Experimental Total	Control Events	Control Total	OR	95%−CI	Weight
Hu, 2020	179	15882	13	6117	5.35	[3.05; 9.41]	51.4%
Molina−Zayas, 2022	4	324	1	103	1.27	[0.14; 11.54]	14.4%
Paixão, 2022	1	155	1	79	0.51	[0.03; 8.21]	9.9%
Felix, 2022	1	72	0	25	1.07	[0.04; 27.11]	7.6%
Zhang, 2022	3	191	0	66	2.47	[0.13; 48.44]	8.8%
Evans, 2023	1	151	0	181	3.62	[0.15; 89.46]	7.8%
**Random effects model**		**16775**		**6571**	**2.76**	**[1.06; 7.17]**	**100.0%**
**Prediction interval**						**[0.31; 24.35]**	

Heterogeneity: $I^2 = 0\%$, $\tau^2 = 0.3779$, $p = 0.43$

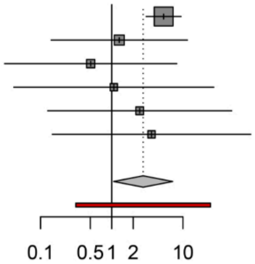

CHEK2

Study	Experimental Events	Experimental Total	Control Events	Control Total	OR	95%−CI	Weight
Hu, 2020	305	15864	23	6101	5.18	[3.39; 7.92]	52.5%
Molina−Zayas, 2022	2	324	1	103	0.63	[0.06; 7.06]	9.4%
Paixão, 2022	2	155	1	79	1.02	[0.09; 11.42]	9.3%
Jin, 2023	1	193	0	190	2.97	[0.12; 73.33]	5.7%
Zhang, 2022	2	191	0	66	1.75	[0.08; 37.02]	6.2%
Evans, 2023	5	151	2	181	3.07	[0.59; 16.03]	17.0%
**Random effects model**		**16878**		**6720**	**3.03**	**[1.36; 6.75]**	**100.0%**
**Prediction interval**						**[0.48; 19.04]**	

Heterogeneity: $I^2 = 1\%$, $\tau^2 = 0.2710$, $p = 0.41$

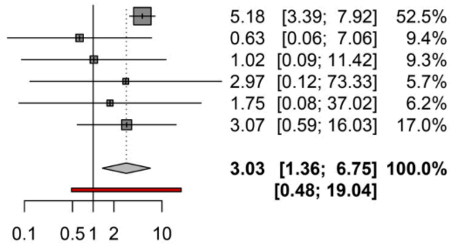

RAD51C

Study	Experimental Events	Experimental Total	Control Events	Control Total	OR	95%−CI	Weight
Hu, 2020	24	14825	26	5680	0.35	[0.20; 0.61]	94.4%
Paixão, 2022	0	155	1	79	0.17	[0.01; 4.18]	2.8%
Zhang, 2022	1	191	0	66	1.05	[0.04; 26.02]	2.8%
**Random effects model**		**15243**		**5850**	**0.36**	**[0.21; 0.61]**	**100.0%**
**Prediction interval**						**[0.01; 11.78]**	

Heterogeneity: $I^2 = 0\%$, $\tau^2 = 0$, $p = 0.72$

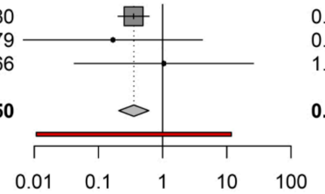

BRIP1

Study	Experimental Events	Experimental Total	Control Events	Control Total	OR	95%−CI	Weight
Hu, 2020	43	14825	26	5680	0.63	[0.39; 1.03]	91.6%
Paixão, 2022	1	155	0	79	1.54	[0.06; 38.33]	2.1%
Felix, 2022	1	72	0	25	1.07	[0.04; 27.11]	2.1%
Zhang, 2022	1	191	0	66	1.05	[0.04; 26.02]	2.1%
Evans, 2023	0	151	1	181	0.40	[0.02; 9.82]	2.1%
**Random effects model**		**15394**		**6031**	**0.65**	**[0.41; 1.04]**	**100.0%**
**Prediction interval**						**[0.31; 1.39]**	

Heterogeneity: $I^2 = 0\%$, $\tau^2 = 0$, $p = 0.97$

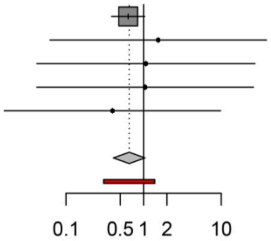

BARD1

Study	Experimental Events	Experimental Total	Control Events	Control Total	OR	95%−CI	Weight
Hu, 2020	24	14778	53	5670	0.17	[0.11; 0.28]	96.2%
Zhang, 2022	1	191	2	66	0.17	[0.02; 1.89]	3.8%
**Random effects model**		**15041**		**5761**	**0.17**	**[0.11; 0.28]**	**100.0%**

Heterogeneity: $I^2 = 0\%$, $\tau^2 = 0$, $p = 0.99$

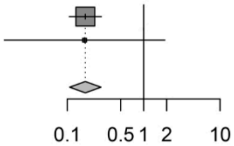

PMS2

Study	Experimental Events	Experimental Total	Control Events	Control Total	OR	95%−CI	Weight
Paixão, 2022	1	155	0	79	1.54	[0.06; 38.33]	17.7%
Zhang, 2022	2	191	0	66	1.75	[0.08; 37.02]	19.7%
Nikitin, 2020	4	172	2	275	3.25	[0.59; 17.94]	62.6%
**Random effects model**		**518**		**420**	**2.52**	**[0.65; 9.75]**	**100.0%**
**Prediction interval**						**[0.00; 16149.28]**	

Heterogeneity: $I^2 = 0\%$, $\tau^2 = 0$, $p = 0.89$

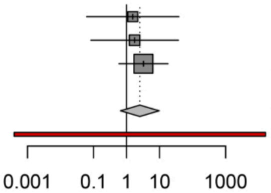

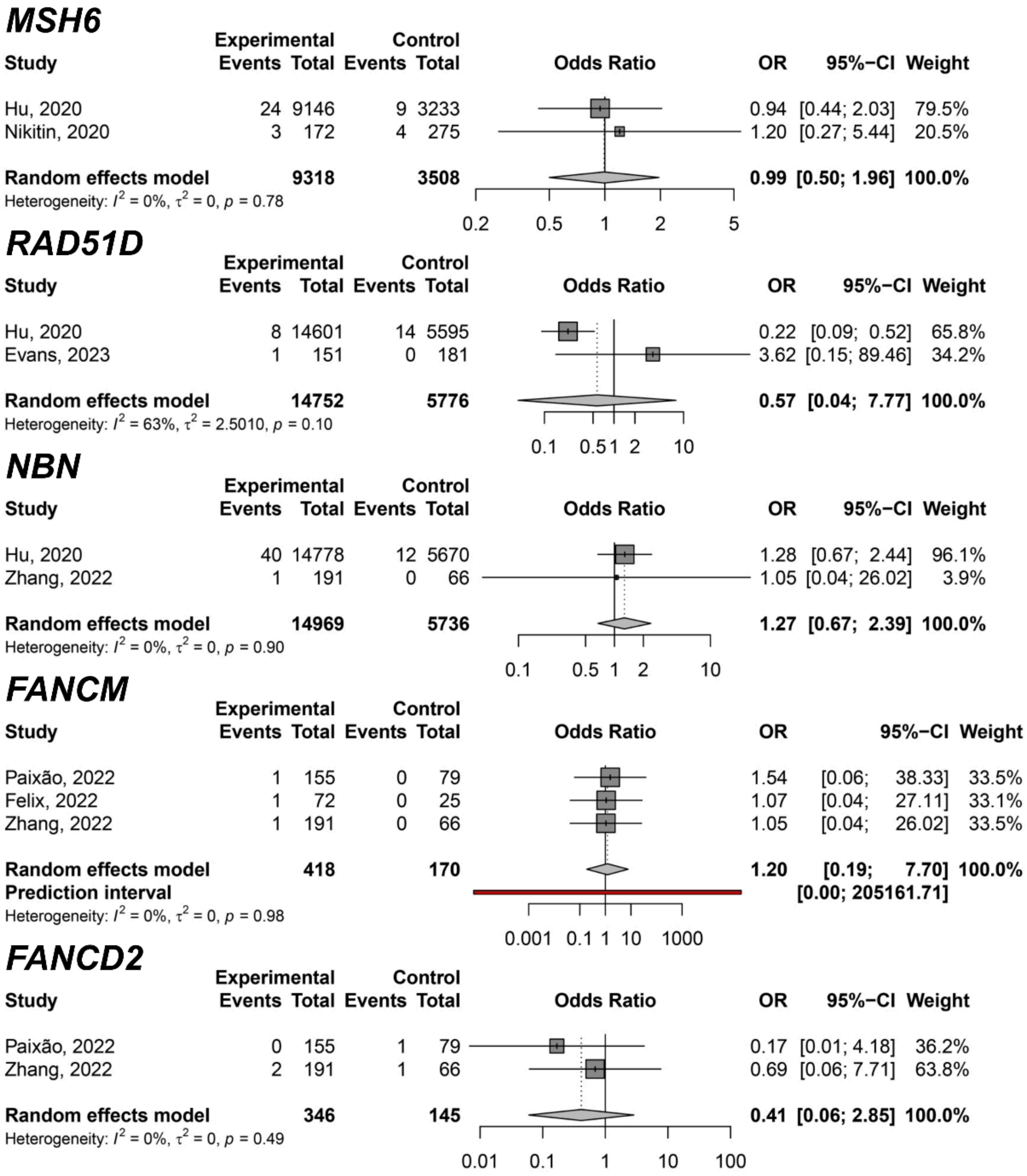

Supplementary figure 10. Forest plots showing the predisposition to HR+HER2- (cases) breast cancer subtype compared to TNBC (controls).
